# Genetic influences on treatment-seeking for common mental health problems in the UK biobank

**DOI:** 10.1016/j.brat.2019.103413

**Published:** 2019-10

**Authors:** Christopher Rayner, Jonathan R.I. Coleman, Kirstin L. Purves, Rosa Cheesman, Christopher Hübel, Helena Gaspar, Kylie Glanville, Georgina Krebs, Genevieve Morneau-Vaillancourt, Gerome Breen, Thalia C. Eley

**Affiliations:** a King's College London, Social, Genetic and Developmental Psychiatry Centre, Institute of Psychiatry, Psychology & Neuroscience, London, UK; b NIHR Biomedical Research Centre, South London and Maudsley NHS Trust, London, UK; c Department of Medical Epidemiology and Biostatistics, Karolinska Institutet, Stockholm, Sweden; d Research Unit on Child Psychosocial Maladjustment, Laval University, Canada

**Keywords:** Treatment-seeking, Treatment gap, Anxiety, Depression, Behaviour, Gene-environment correlation, Heritability, Genetic correlation

## Abstract

The majority of those who experience clinical anxiety and/or depressive symptoms in the population do not receive treatment. Studies investigating inequalities in treatment outcomes rarely consider that individuals respond differently to their experience of the environment. Much of our environment is under genetic influence, via our behaviour, whereby individuals actively select their experiences. If genes influence who seeks and receives treatment, selection bias will confound genomic studies of treatment response. Furthermore, if some individuals are at high genetic risk of needing but not commencing treatment, then greater efforts could be made to engage them. The role of common genetic variation on four lifetime treatment-seeking behaviours (*treatment-seeking, treatment-receipt, self-help, self-medication with alcohol/drugs*) was examined in participants of the UK Biobank (sample size range: 48,106 - 75,322). Treatment-related behaviours were only modestly heritable in these data. Nonetheless, genetic correlations reveal substantial genetic overlap between lifetime treatment-related behaviours and psychiatric disorders, symptoms and behavioural traits. To our knowledge, this is the first study to examine genetic influences on treatment-related behaviours. Further work is required to determine whether genetic factors could be used alongside clinical, social and demographic factors to identify at risk groups and inform strategies which target early intervention.

## Highlights

• Many individuals with anxiety and depression who need treatment, do not receive treatment.
• Personalised medicine is needed to improve treatment access and retention, as well as response.
• Such approaches may benefit from integration of genetic information, alongside social and clinical data.
• We detect genetic overlap between treatment-seeking behaviours and traits of interest, which provides some preliminary insights into treatment-related behaviours and suggests polygenic scores may add predictive value in the future.

## 1 Introduction

One in six adults experience clinical anxiety and/or depressive symptoms in any given week in the UK, but only one in three of those in need of treatment report currently using a treatment (McManus et al., 2016). The disparity between the number of individuals with clinical symptoms and those that receive treatment is called the treatment gap (Kohn, Saxena, Levav, & Saraceno, 2004). A number of studies have estimated that as many as 75% of individuals who have a 12-month diagnosis of depression do not receive treatment (Chisholm et al., 2016; Thornicroft, 2008, 2017). This is despite many having acknowledged a need for treatment, and having contacted services. Some evidence for demographic inequalities in who receives treatment exists. Within the UK, individuals who are White British, female, or middle-aged were more likely to receive treatment than those who were not (McManus et al., 2016). Individuals living in deprived socioeconomic circumstances are more likely to have sought but not actually received treatment (Delgadillo, Asaria, Ali, & Gilbody, 2016; McManus et al., 2016). There are many reasons why individuals might not seek treatment, such as a lack of knowledge to identify clinical symptoms (i.e. poor mental-health literacy), lack of awareness about treatment options, negative beliefs about the effectiveness of treatment, and stigma (i.e., perceived prejudice and/or discrimination against people diagnosed with mental health conditions) (Henderson, Evans-Lacko, and Thornicroft 2013).

Twin studies have shown that a proportion of the variation in nearly all complex human traits can be explained by genetic variation (on average ∼50%; Polderman et al., 2015). This is known as heritability, the proportion of phenotypic variation that can be explained by genetic variation, in a given population at a particular time-point (Visscher, Hill, & Wray, 2008). Analysis of genome-wide genetic data for many traits has shown that a proportion of heritability can be explained by the additive effects of common genetic variants, also known as Single Nucleotide Polymorphisms (SNPs; genetic variation in 1% of the genome, that occurs in >1% of the population). Variation in disorders (e.g. Anxiety and Major Depressive Disorder), personality traits, (e.g. neuroticism, openness, conscientiousness), and life outcomes (e.g. educational attainment) can be explained by common genetic variation, so called SNP heritability (h^2^_SNP_ range: 5-30%; Brainstorm Consortium et al., 2018).

Studies investigating causes of treatment inequalities do not always account for the fact that individuals differ in their response to the environment (Spinath & Bleidorn, 2017). Whether an individual receives treatment, and the type of treatment they receive are components of their environment. Many environmental influences are heritable including experience of stressful life events, social support and marital quality (Kendler & Bake, 2007). Of particular note, the genetic influences on environmental experiences, including stressful life events or social deprivation, overlap considerably with those on traits of interest, such as depression (Clarke et al., 2018; Hill et al., 2016; Thapar, Harold, & McGuffin, 1998). This indicates that genetic variation influences correlations between behaviour and life experiences. In other words, genetic factors influence behaviour, which plays a major role in shaping our environment, so called gene-environment correlation.

One approach towards understanding how genetic influences on different traits relate to one another is to examine their genetic correlation. Exploring genetic correlations allows us to garner information about where genetic influences on the trait of interest (e.g. treatment-seeking) come from. However, understanding what shared genetic variation means is not straightforward, genetic correlations can be interpreted in several ways (Gage, Smith, Ware, Flint, Munafò, & Koifman, 2016; Martin, Taylor, & Lichtenstein, 2018). A genetic correlation may indicate that specific genetic variants influence the two traits independently. Alternatively, in the case where trait A directly influences trait B, any genetic effects on trait A will also influence trait B (Gage et al., 2016). For example, genetic effects might impact on both anxiety symptoms and treatment-seeking. Genes influencing greater symptom severity are likely to influence need for treatment. Genetic variation influencing personality traits, for example openness, might also lead an individual to be more open about their experiences, in turn promoting treatment-seeking. The decision to seek and then commence treatment, is likely to depend in part on genetically influenced individual characteristics, which themselves impact on treatment-seeking behaviours. Knowledge of shared genetic influences between treatment behaviours and relevant traits may help us to understand how genetic influences contribute to treatment outcome inequities. Personalised medicine strategies for anxiety and depression should aim to identify individuals at risk of onset, not receiving treatment, treatment drop-out and of poor treatment outcomes. This information could help clinicians to tailor the whole treatment process to improve patient outcomes. Genetic predictors may add value to prediction models, alongside clinical and socio-demographic data. Personalising courses of antidepressant medication based on genetic data is beginning to show promise towards improving outcomes for patients (Greden et al., 2019).

Whether or not genes influence treatment-seeking should be of particular interest to researchers investigating genetic influences on treatment response. It will be important to identify genetic influences on treatment-seeking and account for them in genomic studies of treatment response. It is also important to consider that only those who seek treatment will receive it, which may have implications for studies and trials investigating treatment efficacy. Those who do not seek treatment (or who seek but do not receive treatment) will be excluded from such analyses, which could bias estimated effects of treatment. For example, belief in the positive effects of treatment has been linked with adherence to treatment and favourable response (Carter et al., 2006; Carter, Crowe, Jennifer, McIntosh, Frampton, & Joyce, 2015; Lambert & Barley, 2001; Ownby, Acevedo, Jacobs, Joshua, & Waldrop-Valverde, 2014). Whereas, negative beliefs about treatment have been associated with reluctance to seek help (Henderson et al., 2013). By not including individuals with negative beliefs about treatment efficacy, the positive effects of treatment might be overestimated. Furthermore, if individuals who need treatment are at high genetic risk of not taking up treatment, then greater efforts could be made to engage them and improve compliance with treatment.

In this study the role of common genetic variation on lifetime treatment-seeking behaviours was examined in a large UK population study, the UK Biobank. Data were available on four treatment-related phenotypes in response to symptoms of anxiety or depression; **treatment-seeking** (*have you ever from sought help from a professional?*), **treatment-receipt** (*have you ever received talking therapy and/or taken prescription medication?*), **self-help** (*have you ever used a structured therapeutic activity and/or over the counter medications to alleviate symptoms?*), and **self-medication with alcohol/drugs** (*have you ever used drugs or alcohol to alleviate symptoms?*). The SNP heritability of these phenotypes was estimated and genetic correlations with psychiatric disorders, symptoms, and behavioural, cognitive and environmental traits were examined. The primary aims were to determine whether different responses to the experience of anxiety and depression symptoms were under genetic influence. Specifically, we examined whether treatment-related phenotypes are heritable and whether any such genetic influences overlap with those on other relevant traits. Where possible analyses were stratified based on whether individuals met diagnostic criteria for a lifetime anxiety or depressive disorder diagnosis. This allowed us to explore the potential role of genetic influences in individuals who did not seek treatment, despite suffering with a high symptom burden. This was contrasted by examining treatment behaviours of those who sought treatment but did not meet criteria for a lifetime diagnosis. This group can be seen as particularly sensitive to their symptoms and perhaps eager to seek help.

## 2 Methods

### 2.1 Sample & phenotypes

The UK Biobank is a UK population study of approximately half a million individuals aged between 40 and 70 (Allen, Sudlow, Peakman, Collins, & Biobank, 2014). The study originally assessed a range of health-related phenotypes and biological measures including genome-wide genotype. More recently a follow-up, online mental health questionnaire was completed by 157,366 participants (Davis et al., 2018). The mental health questionnaire assesses common mental health conditions, including lifetime symptoms of anxiety and depression, and experiences of healthcare. Specifically, participants who reported having worried for a period of six months or longer, having worried more than most people would in a similar situation, having prolonged loss of interest in normal activities, or prolonged feelings of sadness or depression they were asked: “Did you ever tell a professional about these problems? (i.e. medical doctor, psychologist, social worker, counsellor, nurse, clergy, or other helping professional)".

From these data, we defined four treatment-related phenotypes (see supplemental material for sample selection and phenotype definition workflow). **Treatment-seeking** participants reported on seeking help from a professional. **Treatment-receipt** was defined from participants who reported on seeking help and then receiving either prescribed medication or talking therapy for their symptoms. **Self-help** participants reported engaging in a structured therapeutic activity (e.g. mindfulness or yoga) or using over-the-counter medications to help with their symptoms. **Self-medication** was defined from those who reported using alcohol or illicit drugs in response to their symptoms.

For stratified analyses, cases were defined as those who reported sufficient severity and impact of symptoms to meet lifetime criteria for likely DSM-IV for either generalised anxiety disorder or major depressive disorder diagnoses. The **self-report items** were based on questions derived from the Composite International Diagnostic Interview Short Form (CIDI-SF; Davis et al., 2018; Kessler et al., 1998). The CIDI-SF has a classification accuracy of 93% for major depressive disorder and 99% for generalised anxiety disorder (Kessler et al., 1998). Furthermore, major depression and generalised anxiety diagnoses made using the CIDI-SF in the UK Biobank exhibit the same characteristics as would be expected in the general population (Davis et al., 2018). Further information on the validity and reliability of the composite measures of this questionnaire have been published previously, and are detailed elsewhere (Davis et al., 2018; Kessler et al., 1998). Cases were excluded if they self-report lifetime diagnoses of schizophrenia, other psychoses or bipolar disorder. Controls were defined as those not meeting criteria for either anxiety, or depression diagnoses, and were excluded if they reported a diagnosis of any psychiatric disorder. Individuals were excluded from analyses if they did not complete mental health online questionnaire (n = 157,366), or if they did not endorse at least one of the four core common mental health screening items (worried for a period of more than six-months, worry more than most people in a particular situation, loss of interest in usual activities, prolonged feelings of sadness; Max n = 75,322). It should be noted that our 'controls' endorse at least one of the four core common mental health screening items (worried for a period of more than six-months, worry more than most people in a particular situation, loss of interest in usual activities, prolonged feelings of sadness), and as such are not symptom-free. This is a necessary condition for respondents to report on treatment-seeking in the mental health questionnaire.

The whole UK Biobank sample (N∼500,000) were also asked the question: *“Have you ever seen a GP/psychiatrist for nerves, anxiety, tension or depression?“.* From this data, we defined a **formal** treatment-seeking phenotype, i.e. participants who reported on seeking help specifically from their doctor or a psychiatrist. Data were available for 391,213 participants reporting on **formal** treatment-seeking. For these participants, information on symptoms and diagnoses was not available. However, we saw this as an opportunity to test the reproducibility of our primary treatment-seeking analyses in a much larger sample. Results from these analyses are presented in the supplementary material.

### 2.2 Genetic data

Genetic data were drawn from the full release of the UK Biobank data (n = 487,410; Bycroft et al., 2017). Standard quality control, described previously (Coleman et al., 2018) and presented in full in the supplement were applied to the full data. Analyses were limited to common genetic variants imputed to the Haplotype Reference Consortium reference panel with high confidence (McCarthy et al., 2016). Participants were excluded if they had unusual levels of missingness or heterozygosity, they were related to another individual in the dataset, or their phenotypic and genotypic gender information was discordant. All analyses were limited to individuals of White Western European ancestry. This is because 95% of respondents to the mental health questionnaire are of White Western European ancestry. Therefore, we did not have sample sizes required to perform genomic analyses in individuals of different ancestry.

### 2.3 Analyses

All genomic analyses were performed on the residuals from the regression of the binary treatment-related phenotypes and age, sex, six population principal components, batch and assessment centre. Analyses were performed in the whole sample and then stratified by lifetime diagnosis, and also by sex. We performed genome-wide association analyses to estimate the effects of 9.94 million genome-wide genetic variants on each phenotype (BGENIE v1.2; Bycroft et al., 2017). We then estimated how much phenotypic variance could be explained by the additive effects of common genome-wide genetic variants, so called, SNP heritability (h^2^_SNP_). We calculated SNP heritability using linkage disequilibrium (LD) score regression (Bulik-Sullivan et al., 2015). A non-technical explanation of the principles underlying LD score regression is provided in the Supplement. As all of the phenotypes examined were binary, observed SNP heritability estimates were converted to the liability scale at the full range of population prevalence estimates. SNP heritability estimates calculated using LD score regression are limited to the additive effect of genetic variation captured by common variants analysed in GWAS. As such, SNP heritability does not account for the influence of other genetic factors, such as dominant, recessive or interaction effects, rarer genetic variants (population frequency < 5%), nor other types of genetic variation (e.g. copy number variants). Therefore, SNP heritability is an estimate of the lower bound of phenotypic variance that can be explained by genetic variance, and the upper bound of phenotypic variance that can be predicted by polygenic scores derived from the genotype data.

It is also important to note that the relative contributions of genetic and environmental factors to a phenotype are population (or sample) specific. Variance in genetics, variance due to environmental factors, and the correlation between genes and environment differs between populations (and samples), and is subject to change over time. As such, sampling bias also attenuates heritability estimates.

We also estimated genetic correlations (r_g_) between treatment-related phenotypes and also with a range of psychiatric disorders, personality and behavioural traits. A genetic correlation estimated using LD score regression is simply the correlation between the SNP effects for a pair of phenotypes. As such, the genetic correlations estimated are the proportion of common genetic (SNP) effects that are shared between two phenotypes. We selected a range of well-powered GWAS of psychiatric, behavioural and related traits to provide a thorough examination of the genetic overlap between treatment-related phenotypes and behaviour, psychopathology (for the full list, including references see supplement and/or Fig. 2). Significance was assessed after Bonferroni multiple testing correction.

## 3 Results

### 3.1 Phenotype distribution

Phenotype and genotype data were available for participants reporting on treatment-seeking *(n=71,416)*, treatment-receipt (*n=48,106)*, self-help (*n=75,322)*, and self-medication (*n=75,128;* Table 1*)*. Based on the self-report items, the lifetime prevalence of generalised anxiety disorder and major depressive disorder was 11% and 38% respectively (in individuals seeking treatment). Combined, the prevalence of experiencing a common mental disorder across the lifespan was 41%. All participants in our primary sample reported experiencing at least one core symptom of anxiety or depression across their lifetime. The majority (67%) of the sample reported informing a professional about their symptoms and the majority of these individuals (84%) reported receiving treatment for their symptoms (56% of the total sample). Of the whole sample, 20% report using a self-help approach such as exercise, mindfulness or over-the-counter medication, and 15% report self-medicating with alcohol or drugs (for the overlap between these treatment-seeking phenotypes, see Table 2).

**Table 1:** Treatment-seeking phenotype distributions in the full sample and stratified by CIDI derived lifetime diagnosis of a common mental disorder status and sex.

		*N*^*(a)*^	Lifetime diagnosis		Sex	
			*Controls*^*(b)*^	*Cases*^*(b)*^	*Female*^*(b)*^	*Male*^*(b)*^
Treatment-seeking	***No***	23,362	16,789	4,222	12,528	10,834
	***Yes***	48,054	14,700	25,810	32,706	15,348
	***Prev.***^(∗)^	0.67	0.47	0.86	0.72	0.59
Treatment-receipt	***No***	7,670	4,005	2,552	4,914	2,756
	***Yes***	40,436	10,715	23,283	27,829	12,607
	***Prev.***^(∗)^	0.84	0.73	0.9	0.85	0.82
Self-help	***No***	60,091	29,451	22,515	36,132	23,959
	***Yes***	15,231	4,317	8,519	11,587	3,644
	***Prev.***^(∗)^	0.2	0.13	0.27	0.24	0.13
Self-medication with alcohol/drugs	***No***	64,171	30,642	24,714	41,670	22,501
	***Yes***	10,957	3,042	6,252	5,924	5,033
	***Prev.***^(∗)^	0.15	0.09	0.2	0.12	0.18

*Proportion of individuals that endorse each phenotype in (a) individuals who report on the phenotype, drawn from the full sample and (b) individuals who report on the phenotype, drawn from each strata; No = never in their lifetime; Yes = at least once in their lifetime.

**Table 2:** Overlap between treatment-seeking phenotypes.

		Treatment-seeking ^*(i)*^			Treatment-receipt ^*(i)*^			Self-help ^*(i)*^		
		*No*	*Yes*	%^*(a)*^	*No*	*Yes*	%^*(a)*^	*No*	*Yes*	%^*(a)*^
Treatment-receipt ^*(ii)*^	***No***	0	7,665	100						
	***Yes***	0	40,389	100						
	**%**^*(b)*^	–	84							
Self-help ^*(ii)*^	***No***	20,831	35,971	63.3	6,100	29,916	83.1			
	***Yes***	2,521	12,056	82.7	1,557	10,506	87.1			
	**%**^*(b)*^	10.8	25.1		20.3	26				
Self-medication with alcohol/drugs ^*(ii)*^	***No***	20,517	40,324	66.3	6,684	33,685	83.4	52,453	11,708	18.2
	***Yes***	2,772	7,603	73.3	933	6,677	87.7	7,467	3,488	31.8
	**%**^*(b)*^	11.9	15.9		12.2	16.5		12.5	23	

**(a)** % of individuals who endorse phenotype (i) out of the total number of individuals who either have never endorsed (0) or have endorsed (1) phenotype (ii); **(b)** % of individuals who endorse phenotype (ii) out of the total number of individuals who either (0) have never endorsed or (1) have endorsed phenotype (i); No = never in their lifetime; Yes = at least once in their lifetime

### 3.2 Heritability analyses

**Fig. 1:**
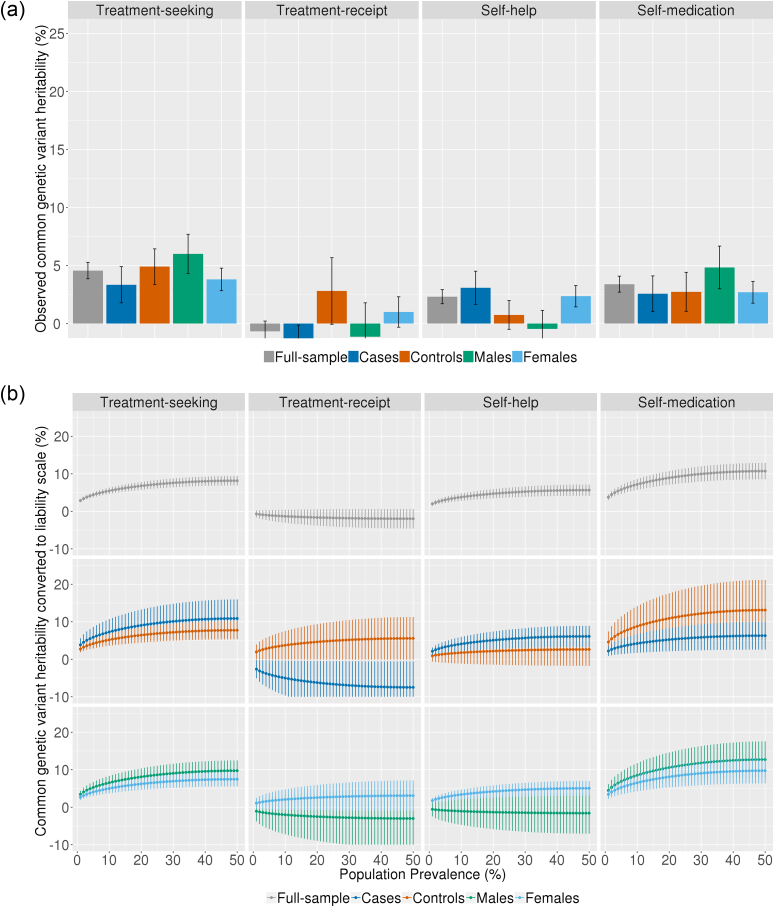
(a) Observed common genetic variant heritability estimates of treatment-seeking phenotypes in the whole cohort, and stratified by case/control status and sex; (b) Common genetic variant heritability curves: heritability estimates of treatment-seeking phenotypes converted to the liability scale at the full range of population prevalence.

Our analyses indicate that treatment-seeking is modestly heritable. In the whole cohort we detect small, but significant observed estimates of SNP heritability for treatment-seeking (h^2^_*SNP*_ = 3.9% se=.7%), self-medication (h^2^_*SNP*_ = 3.4% se=.7%) and self-help (h^2^_*SNP*_ = 2% se=.6%; Fig. 1a). The heritability of receiving treatment (as opposed to seeking treatment but not receiving it) was smaller than our analysis was powered to detect (80% power to detect h^2^_*SNP*_*=*4.5%; 70% power to detect h^2^_*SNP*_ *=*4%; Hemani & Yang, 2017).

**Fig. 2:**
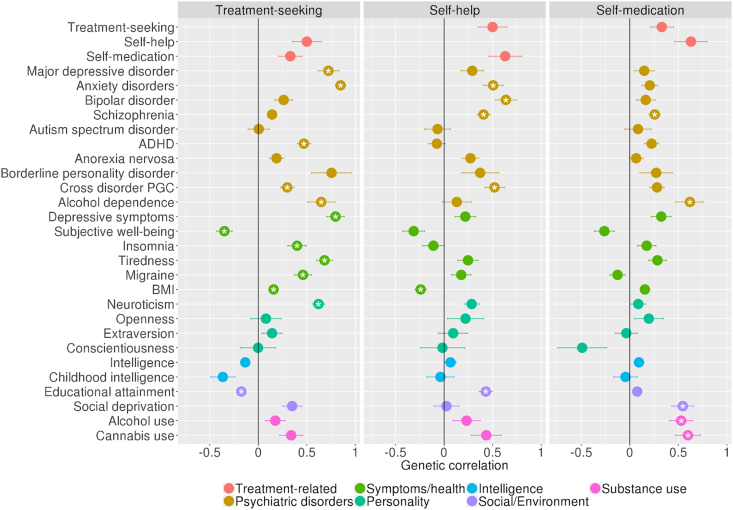
Genetic correlations between the treatment seeking phenotypes and psychiatric, and behavioural traits.

We also estimated the heritability of all the treatment-related phenotypes in cases and controls, and in males and females separately (Fig. 1a). However, in these smaller sub samples, the estimates are small and standard errors are large and overlap substantially. This could suggests that the estimates are not significantly different between strata (cases/controls; males/females). It is more likely that the study was underpowered to detect subtle differences.

The lifetime prevalences of treatment-related phenotypes are difficult to estimate, so we converted estimates to the liability scale across the full range of population prevalence (Fig. 1b). Heritability on the liability scale ranges from 2% to 9% for treatment-seeking, 2% to 5% for self-medication and 1% to 6% for self-help. Again, the standard errors of the heritability estimates overlap substantially when the analyses are stratified by case-control status.

### 3.3 Genetic correlations

Treatment-seeking, self-help and self-medication all show modest genetic correlations with one another, that are not significant after multiple testing correction. Treatment-seeking has genetic correlations with both self-medication (r_g_ = .36, se=.13) and self-help (r_g_=.52, se=.15). Self-medication has a genetic correlation with self-help (r_g_=.65, se=.17). The largest genetic correlations were observed between treatment-seeking and anxiety disorders (r_g_=.85, se=.05) and major depression (r_g_=.72, se=.11) (Fig. 2). Self-help has positive genetic correlations with educational attainment (r_g_ = .43, se=.07), schizophrenia(r_g_=.41, se=.07), bipolar disorder (r_g_=.64, se=.11), cross disorder psychopathology (r_g_=.52, se=.10), anxiety disorders (r_g_=.51, se=.10) (and a negative genetic correlation with body mass index (r_g_ = -.24, se=.06). Self-medication has significant positive genetic correlations with social deprivation (r_g_=.55, se=.13), cannabis use (r_g_=.60, se=.13), schizophrenia (r_g_=.26, se=.06), alcohol use (r_g_=.53, se=.12) and alcohol dependence (r_g_=.62, se=.14) (references for GWAS summary statistics used to calculate genetic correlations are detailed in the supplementary material).

To understand more about the behavioural underpinnings of treatment-seeking, analyses were stratified based on reported symptom burden (i.e. CIDI-SF derived anxiety/depression caseness). Here, the genetic contributions to treatment-seeking in participants who reported high symptom burden was compared to those influencing participants who reported low symptom burden. However, stratified analyses resulted in reduced statistical power to detect genetic correlations after corrections for multiple testing. Estimates were not significantly different between the strata and standard errors overlap substantially (see Supplementary Figure 5).

As a final step, we performed supplementary analyses using data available from the remaining UK Biobank sample participants, which assessed **formal** treatment-seeking. This terminology refers to when someone has specifically sought treatment (in this case for nerves, anxiety, tension or depression) from a medical professional, i.e. a GP or psychiatrist. Further details including results are presented in the supplemental material.

## 4 Discussion

To our knowledge, this is the first study to use genomic data to examine genetic influences on treatment-seeking and related phenotypes. We utilised participant responses to the UK Biobank mental health questionnaire (MHQ), which assessed adults for lifetime mental health, and their responses to their own mental health symptoms. Responses included seeking-treatment, receiving treatment (prescribed medications or psychological therapy), self-help (obtaining non-prescribed medications or enrolling in a therapeutic activity), or self-medicating with alcohol and/or drugs.

Of the UK Biobank participants who completed the MHQ and who endorse at least one symptom of anxiety/depression (necessary for inclusion), the lifetime prevalence of anxiety and/or depression was 41%. More than 85% of those who met lifetime criteria for a diagnosis, sought help, and 90% of those who sought help, received treatment. Of note, 47% of individuals who have experienced an anxiety/depression symptom seek help, even though they do not necessarily meet criteria for a clinical diagnosis. Furthermore, 73% of these individuals go on to receive treatment.

The treatment gap estimated in this sample is thus much lower than previous point prevalence estimates. Previous estimates from the UK suggest that at any one point in time, only ∼30% of individuals are receiving the treatment they need (McManus et al., 2016; Thornicroft et al., 2017). There are several reasons why rates of treatment are much higher in this UK biobank sample than in previous studies. Firstly, our estimate reflects lifetime prevalence of treatment-seeking and treatment-receipt rather than the point prevalence estimates provided elsewhere. Of course individuals are more likely to have received treatment at some point in their lives than in the context of any single episode. As such, these estimates (90% lifetime prevalence of treatment-receipt; ∼30% point prevalence of treatment-receipt) are not directly comparable.

The high rates of treatment-seeking and receipt in this UK Biobank study may also be related to other aspects of the study design. All participants in these analyses endorsed at least one of the four core common mental health symptom screening items, and as such are not symptom-free. Moreover, the UK Biobank sample (particularly the MHQ respondent sub-sample) has more individuals from communities with more advantaged socioeconomic circumstances, and with higher educational attainment, than the general UK population (Davis et al., 2018). These characteristics are also associated with greater access to treatment (Delgadillo et al., 2016). Furthermore, participants with greater mental health burden may have been lost to attrition. Genomic analyses of UK Biobank participants who did not provide details for recontact (i.e. further participation in the study) and those who did not respond to the mental health questionnaire, reveals that level of participation is a heritable trait (h^2^_SNP_ = 8-10%) (Adams et al., 2019). Furthermore, non-participation is positively genetically correlated with a range of mental disorders and physical diseases.

Our goal was to determine whether lifetime treatment-seeking behaviour was influenced by common genetic variation, and if so, whether treatment-seeking shared genetic influences with psychiatric disorders and behavioural traits. Our analyses indicate that treatment-seeking, self-medicating with alcohol/drugs and self-help in response to symptoms of anxiety or depression are only modestly heritable phenotypes in these data. We used linkage disequilibrium score regression to estimate heritability and genetic correlations because it is less computationally demanding than other methods and enables the calculation of genetic correlations with external traits (such as the psychiatric disorders and behavioural traits included herein). However, it is likely that LD score regression underestimates heritability, relative to other contemporary approaches (Evans et al., 2017; Ni et al., 2018). It is also important to note that SNP heritability estimates only consider common genetic variation, rather than the whole genome. Twin heritability estimates, which capture the proportion of variation in a trait due to all genetic influences are usually at least twice that of SNP heritability estimates (Polderman et al., 2015; Brainstorm Consortium et al., 2018). For example, neuroticism has a twin heritability of ∼50% (Boomsma et al., 2018) but a SNP heritability of ∼10% (Nagel et al., 2018). As such, our estimates are likely to represent the lower bounds of heritability for treatment-seeking and related traits. Furthermore, the SNP heritability estimate provides an upper limit to the prediction of a phenotype using polygenic scores. The SNP heritability estimates of both treatment-seeking and self-medication with alcohol/drugs in this sample range from 2% to ∼10% (on the liability scale). As such, this would be the upper limit for genomic prediction of these phenotypes in this sample, when using common genetic variation. Nonetheless, the upper limit of our estimate is 10%, which is a substantial amount of variance in terms of prediction, when combined with other similarly powerful independent predictors. However, we do not model prediction in this present study.

There was, however, substantial genetic overlap between treatment-seeking and both anxiety and depression. Furthermore, the high genetic correlations with the other phenotypes examined reflect a similar pattern of genetic correlations to that observed in genomic studies of depression and anxiety disorders themselves (Brainstorm Consortium et al., 2018). This suggests that the genetic influences on treatment-seeking for anxiety and depression are largely the same as those influencing the experience of these disorders. Indeed, one might expect that the primary genetic influences on treatment-seeking are related to symptom severity. Nonetheless, many individuals who report high levels of symptom burden, have never sought treatment. In order to try and examine treatment-seeking as a behaviour independent of symptom experience, analyses were stratified by probable case status. As such, individuals who report high levels of symptoms, who probably needed treatment but never sought it, were contrasted with individuals who reported experience of mild symptoms and sought treatment anyway. We found no significant differences between these two strata. However, by halving the sample size, statistical power was attenuated and any subtle differences were not detected.

We also examined genetic influences on self-help and on self-medication (-with alcohol/drugs). Self-help shows a similarly wide set of genetic correlations, though fewer reach statistical significance. Self-help is positively genetically correlated with anxiety disorders, bipolar disorder, schizophrenia, and general psychopathology, as well as favourable educational outcomes. There is also a negative genetic correlation with BMI.

Firstly, the BMI finding suggests that a portion of the genetic effects that promote self-help behaviour, also contribute to lower BMI. It makes sense that self-help behaviours are related to healthy lifestyle habits (exercise, diet etc) and thus, lower BMI. There is also the possibility that many of the respondents use exercise as a therapeutic self-help activity as a way to deal with symptoms. The positive genetic correlations between self-help and and educational outcomes is also of interest, especially when considering the previous self-help-BMI genetic correlation. Phenotypically, greater educational attainment is associated with participation in regular exercise (Trost, Owen, Bauman, Sallis, & Brown, 2002). Genetic effects that motivate people to self-help, may also motivate them to succeed with their education. The genetic links between self-help and education could feasibly be linked to personality traits such as openness to experience (von Stumm, 2018) or self-efficacy (Choi et al., 2019), which are associated with favourable educational outcomes. However, we find no evidence of a genetic relationship between openness and self-help from our analysis. Alternatively, genetic effects may influence behaviours to promote further education, which improves mental health literacy, promoting the use of self-help activities. Here, education is on the causal pathway to self-help. However, we can not infer direction of causality from these data.

The self-help phenotype includes participants who report enrolling in mindfulness based activities. To date, evidence in support of self-help for the treatment of anxiety and depression is mixed. Mindfulness based interventions have been shown to have small effects, especially when compared to placebo (Hedman-Lagerlöf, Hedman-Lagerlöf, & Öst, 2018). However, evidence from an observational study in 1.2m individuals with a lifetime diagnosis of depression, highlights the association between different types of exercise (including mindfulness-based exercises) and better mental health (Chekroud et al., 2018). While these associations do not necessarily support the use of exercise or mindfulness as an intervention, findings do indicate that on average, people who are susceptible to depression, who exercise and/or engage in self-help like activities, report lower levels of stress than those who do not. This present study also examines observational data. Therefore, we cannot determine whether self-help causes a reduction in symptoms. Paradoxically, self-help is also genetically correlated with bipolar-disorder and schizophrenia, both of which also have positive genetic correlations with educational attainment. The genetic correlations between self-help and both education and schizophrenia remain significant in individuals who meet diagnostic criteria, but not in sub-diagnostic-threshold individuals. Previous research published from UK Biobank data shows that participants with higher propensity for psychotic symptoms over-report their level of physical activity (Firth et al., 2018). The genetic links between psychotic/manic symptoms and self-help are also difficult to disentangle. One hypothetical explanation might be that over-reporting of (and perhaps even enrollment in) certain self-help activities (as “alternative medicine” strategies) might be linked to increased genetic risk for symptoms such as delusions.

Self-medication is significantly genetically correlated with schizophrenia, alcohol dependence, alcohol use and cannabis use. The strong weighting towards genetic correlations with measures of substance use is expected, due to the phenotypic similarity. Of particular interest, self-medication has a genetic correlation with social deprivation (r_g_ = .55; se=.11). Genes influencing self-medication with drugs or alcohol thus also tend to be associated with social deprivation. While the genetic correlation between treatment-seeking and social deprivation was not statistically significant after correction for multiple testing (r_g_ = 0.35, se = 0.1, p = 0.0005; Bonferroni p-value <0.0001), it is notable that in supplementary analyses, we detected a significant genetic correlation between **formal** treatment-seeking (i.e. specifically seeking treatment from a GP or psychiatrist, see supplemental material) and social deprivation (r_g_ = 0.44, se = 0.06, p = 3.80E-15). The genetic correlation between **broad** treatment-seeking (primary analysis) and **formal** treatment-seeking (supplementary analysis) is not significantly different from 1 (r_g_ = 0.99, se = 0.08, p = 8.84E-30). While the effect in the primary analyses does not meet strict criteria for statistical significance, the replication of this finding is interesting and worthy of further discussion. The genomic correlations observed here reflect a greater genetic propensity for both the experience of symptoms, treatment-seeking (given a need) and self-medication, in individuals who live in more socially deprived areas. Indeed, genetic influences on depression have greater effects in socially deprived areas (Strachan, Duncan, Horn, & Turkheimer, 2017). Multiple epidemiological studies now indicate that there is greater demand for, but poorer access to treatment in socially deprived areas (Delgadillo et al., 2016), known as the ‘inverse care law’ (Saxon et al., 2007). Furthermore, income inequality is also associated with increased burden of mental illness related morbidity (Ribeiro et al., 2017), greater stigmatization and alcohol and drug use (Kulesza, Watkins, Ober, Osilla, & Ewing, 2017; Room, 2005). Individuals who self-medicate in response to symptoms of depression are at greater risk of developing alcohol dependence (Crum et al., 2013) and substance use disorder (Turner, Mota, Bolton, & Sareen, 2018). Of note, risk factors for self-medication include being male, younger, Caucasian and/or separated from a partner, whereas, evidence of associations with education and income is mixed (Turner et al., 2018). The relationship between area-level deprivation and health-related outcomes is complex and likely to be confounded by individual level characteristics (Karriker-Jaffe, 2011). Further phenotypic analyses are required to disentangle social factors associated with treatment-seeking, treatment-receipt and self-medication with alcohol and drugs.

The interpretations of these findings should be considered with several limitations in mind. First, all analyses were performed using data from a population-based sample. As noted earlier the sample has higher average socioeconomic circumstances than the general population, potentially reducing generalisability of the findings. Non-participation in the UK Biobank is associated with greater genetic burden for a range of mental disorders and physical diseases (Adams et al., 2019). It is therefore possible that there are treatment-seeking differences between those who took part in the study compared to the general population. While the prevalence estimates are not generalisable, factors associated with the phenotypes may be more so. Multiple studies exploring risk factor for diseases using the UK Biobank sample have detected associations that were expected and supported by findings from elsewhere (for example: Ganna and Ingelsson 2015). Second, all data analysed were retrospective self-report, which brings its own biases. It is likely that participants both under- and over-report their past experience of symptoms, as well as their lifetime experiences of healthcare. Thus we are unable to draw strong conclusions regarding genetic influence on treatment-seeking at a given point in time (i.e. specifically at the time of need). Future research on this topic would ideally identify groups of individuals with current symptoms, and examine concurrent treatment-seeking attitudes and behaviours including both those receiving and not receiving treatment. This would enable a more precise estimation of the role of genetic factors with respect to accessing and receiving treatment at the point of need. Such data would provide a better indication of whether genetic predictors might be useful in models which guide early intervention/outreach strategies. Finally, with regard to the analytical approach, when the heritability of a trait is modest, estimates of genetic correlation can be inflated. As such, these estimates should be interpreted with caution.

In conclusion, treatment-seeking behaviours are modestly heritable in these data. Statistically significant genetic correlations provide preliminary insights on the genetic architecture of these treatment-related behaviours. While the relationship between these traits might seem obvious, it is encouraging that genetic correlations recapitulate relationships expected from phenotypic observations. This demonstrates the utility of genomics, to help identify shared genetic risk between phenotypes and even highlight phenotypic correlations, which may otherwise be difficult to measure form individual level data collected. Where data on a wide range of outcomes is unavailable, genetic data can be used to examine overlap between phenotypes in the population. Further work is required to understand the shared and the unique genetic influences on treatment-related behaviours and relevant phenotypes, and to understand the causal relationships between them. This could be facilitated by novel genomic methods, which allow for the combined analyses of multiple traits (Grotzinger, Rhemtulla, de Vlaming, & Ritchie, 2018; Zhu et al., 2018) and causal inference (Smith et al., 2005). Given that treatment-seeking is heritable, it will be necessary for genomic studies of treatment response (i.e pharmacogenetics and therapygenetics) to account and adjust for genetic influences on treatment-seeking in order to delineate the biological mechanisms of treatment response. In other words, when identifying genes that influence outcomes following treatment, it will be important to take into account genetic influences on treatment-seeking so that these are not incorrectly thought to influence outcome. More work is needed to work out how best to make such an adjustment. Finally, our findings suggest that there is shared genetic risk for social deprivation with both treatment-seeking, and self-medication with drugs/alcohol. While genetics can be used to highlight behaviours and environments that share genetic risk and may add value to predictive models, work investigating social, demographic or clinical barriers to treatment will also be required if we hope to identify individuals at risk of not receiving timely treatment. Most work focusing on personalising treatments for anxiety/depression is concerned with predicting outcomes following treatment. This is because only approximately half of those who receive treatment achieve remission. However, only a third of those with clinical symptoms receive treatment in the first place (McManus et al., 2016). As such, identifying barriers to treatment, and promoting early intervention could be a more promising avenue towards reducing the morbidity of anxiety and depressive disorders. To date, prediction models of treatment outcomes have been unsuccessful at predicting outcomes with clinical utility. This is likely to do with the complex network of factors that influence treatment outcomes. Many factors will be difficult to account for. However, genetic data may provide another level of assessment that can complement traditional clinical assessment, and may also provide opportunities to control for individual characteristics that are otherwise unmeasured. This work is in its infancy and while we agree that genetics will not predict outcomes alone, it has the potential to add value in combination with clinical and demographic predictors. Future work would benefit from prospective and longitudinal study designs, collecting detailed clinical, social, demographic and genetic data. Such data could enable personalised medicine initiatives to predict need-for-treatment and guide assertive outreach interventions towards those that are unlikely to seek and/or receive treatment.

## Conflicts of interest

The authors declare no conflict of interest.
