# Supplementary material for: Genetic influences on treatment-seeking for common mental health problems in the UK biobank
Source: Behav Res Ther. 2019 Oct;121:103413. doi: 10.1016/j.brat.2019.103413 (PMC6873796; doi:10.1016/j.brat.2019.103413)
Supplement: Multimedia component 1 [file mmc1.docx]

Supplementary Material for BRAT: Genetic influences on treatment-seeking for common mental health problems in the UK Biobank

**Supplementary Methods**

[***Sample selection & phenotype definition***](#_716f392vi0d4) ***2***

[***Genotype Quality Control***](#_wcxeubfof6no) ***3***

[***Genome Wide Association Studies (GWAS)***](#_vmtab7tt9hr2) ***4***

[***Linkage disequilibrium score regression***](#_y7vmx5iiwyu8) ***4***

[***Comparing heritability estimates and genetic correlations using block jackknife and LD Score***](#_hy52it3fgrmp) ***5***

[***Full list of GWAS traits used for genetic correlations (with reference)***](#_7nsdgpqu0c99) ***6***

**Supplementary Results**

[***LD score regression SNP heritability and intercept estimates***](#_nn5is031z9p) ***7***

[*Supplementary Table 1. LD score regression SNP heritability and intercept estimates, and standard errors from analyses in the full sample and from stratified analyses*](#_k6rprebbvwpc) *7*

[***Manhattan and QQ plots of genome-wide associtation p-values, for analyses in the full sample***](#_3as7aqr0i31t) ***8***

[*Supplementary Figure 1. Manhattan and QQ plots for GWAS of treatment-seeking in the full sample*](#_oosy1htevg1a) *8*

[*Supplementary Figure 2. Manhattan and QQ plots for GWAS of treatment-receipt in the full sample*](#_d7cdppd54bsd) *8*

[*Supplementary Figure 3. Manhattan and QQ plots for GWAS of Self-medication in the full sample*](#_ce76wa7i3ymr) *9*

[*Supplementary Figure 4. Manhattan and QQ plots for GWAS of Self-help in the full sample*](#_qbkrrzyx1wxf) *9*

[*Supplementary Figure 5. Genetic correlations of treatment phenotypes and external traits in the “case/control” stratified samples*](#_gjv11nf2ckyv) *10*

[*Supplementary Figure 6. Genetic correlations of treatment phenotypes and external traits in the sex stratified samples*](#_ithnkx5qhujq) *11*

[*Supplementary table 2. Genetic correlations between treatment phenotypes and external traits (psychiatric disorders, behavioural traits; ordered by significance)*](#_x3zimic4ug4v) *11*

[*Supplementary table 3. Internal genetic correlations between treatment seeking phenotypes*](#_5wpwloqdt793) *17*

[***Formal treatment-seeking (replication analyses)***](#_1209oymx75z5) ***20***

[*Supplementary table 4. Phenotype distribution of primary treatment-seeking phenotype and supplementary, formal treatment-seeking phenotype*](#_kr4gvuporqxm) *20*

[*Supplementary Figure 7*](#_neu8xw7fckr1)

[*(a) Observed common genetic variant heritability estimates of treatment-seeking in the primary (broad) and secondary cohort (formal), and stratified by sex*](#_neu8xw7fckr1)

[*(b) Common genetic variant heritability curves: heritability estimates of treatment-seeking converted to the liability scale*](#_neu8xw7fckr1) *21*

[*Supplementary Figure 8. Genetic correlations of treatment-seeking and external traits stratified by sex in the primary sample (broad treatment-seeking) and the secondary sample (formal treatment-seeking)*](#_pkqukr1kltw5) *23*

[*Supplementary Figure 9. Manhattan and QQ plots for GWAS of Formal treatment-seeking*](#_m7i10txfqput) *23*

[***Supplementary References***](#_82iwid53e6uo) ***25***

Supplementary Methods

# Sample selection & phenotype definition


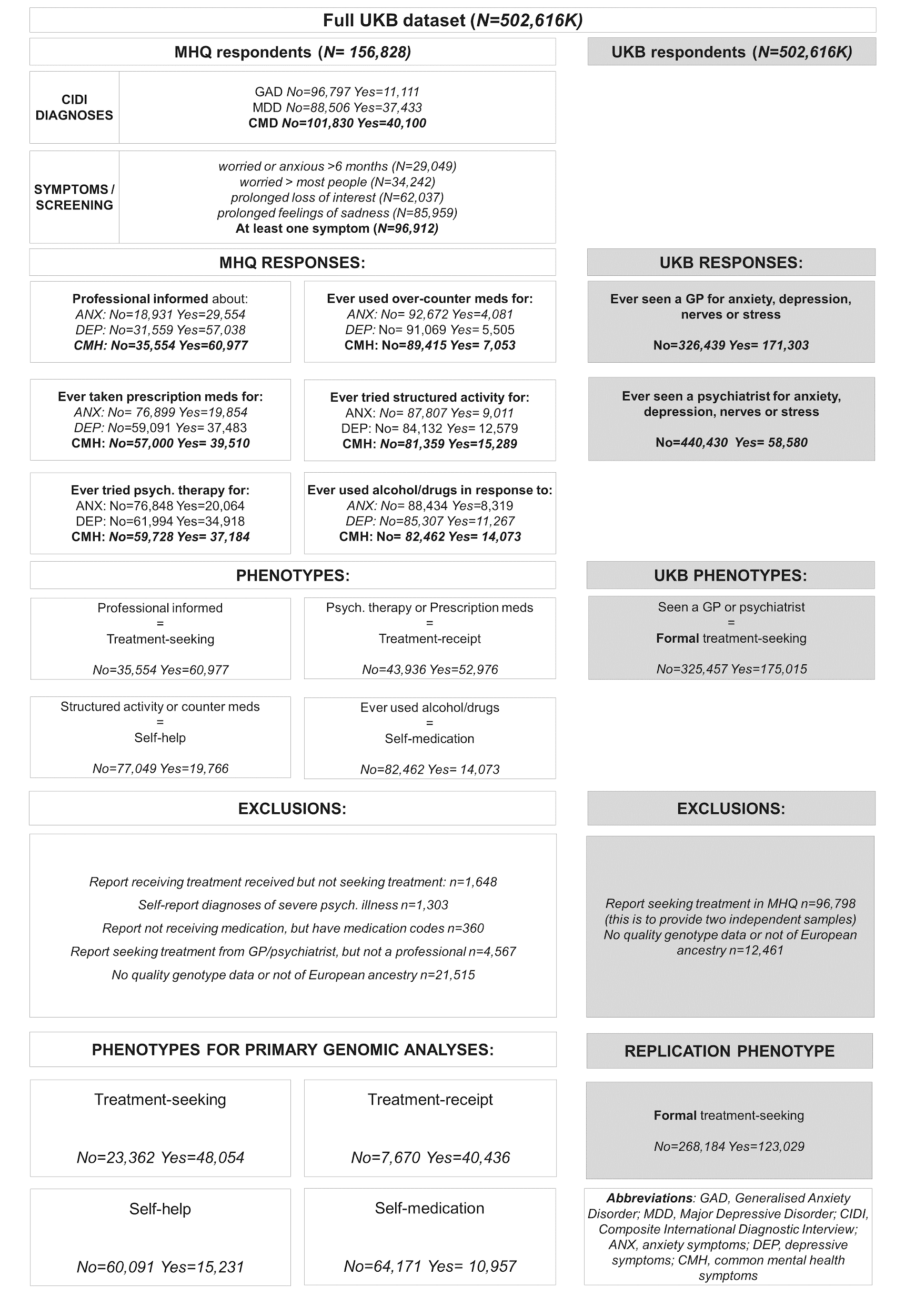


# Genotype Quality Control

Genetic data for GWAS analyses came from the full release of the UK Biobank data (N=487,410; [(Allen et al. 2014)](https://paperpile.com/c/ir95Fc/xuln). Autosomal genotype data from two highly-overlapping custom genotyping arrays (covering ~800,000 markers) underwent centralised quality control before being imputed in a two-stage imputation to the Haplotype Reference Consortium (HRC) and UK10K (for rarer variants not present in the HRC) reference panels [(Bycroft et al. 2017; McCarthy et al. 2016; UK10K Consortium et al. 2015)](https://paperpile.com/c/ir95Fc/WIdz+iXWO+DIfJ).

In addition to this central quality control, variants for analysis were limited to common variants (minor allele frequency > 0.01) that were either directly genotyped or imputed from the HRC with high confidence (IMPUTE INFO metric > 0.4) [(McCarthy et al. 2016)](https://paperpile.com/c/ir95Fc/iXWO).

Individuals were excluded where recommended by the UK Biobank core analysis team for unusual levels of missingness or heterozygosity, or if they had withdrawn consent for analysis. Using the genotyped SNPs, individuals with call rate < 98%, who were related to another individual in the dataset (KING r < 0.044, equivalent to removing up third-degree relatives and closer; [(Manichaikul et al. 2010)](https://paperpile.com/c/ir95Fc/NSdB)) or whose phenotypic and genotypic gender information was discordant (X-chromosome homozygosity (F_X_) < 0.9 for phenotypic males, F_X_ > 0.5 for phenotypic females) were also excluded. Removal of relatives was performed using a "greedy" algorithm, which minimises exclusions (for example, by excluding the child in a mother-father-child trio). All analyses were limited to individuals of European ancestry, as defined by 4-means clustering on the first two genetic principal components provided by the UK Biobank [(Warren et al. 2017)](https://paperpile.com/c/ir95Fc/AbCu). This ancestry group included 95% of the respondents to the mental health questionnaire - as such, the non-European ancestry groups were considered too small to analyse informatively. Principal components analysis was also performed on the European-only subset of the data using the software flashpca2 [(Abraham, Qiu, and Inouye 2017)](https://paperpile.com/c/ir95Fc/ykhr).

After quality control, individuals with high-quality genotype data and who had completed the online mental health questionnaire were retained for analysis (N=126,522). Variants for these analyses were limited to common variants (minor allele frequency > 0.01) with call rate >98% that were in approximate Hardy-Weinberg equilibrium (HWE test p > 10-8). The same individuals were used for analyses using the imputed and the genotyped data.

# Genome Wide Association Studies (GWAS)

Genome-wide association analyses were performed via linear regression in BGenie v1.2 [(Bycroft et al. 2017)](https://paperpile.com/c/ir95Fc/WIdz). First, treatment-related phenotypes were residualised for age, sex and six population principal components (to control for population stratification). Residualised phenotypes were then regressed on the number of reference allele copies (0, 1 or 2) at 9.94 million genotypes.

# Linkage disequilibrium score regression

Linkage disequilibrium (LD) is the non-random association of DNA sequence variants in specific regions of the genome in a given population. The LD score for each genetic variant is calculated as the sum of its Pearson’s correlation coefficients with all other measured variants within a one megabase window (a region of 10^6^ DNA bases). Genome-wide association effect size estimates for each variant are confounded by the effects of all variants with which it is correlated. A ‘non-causal’ variant that is correlated with a causal variant will have a higher association test statistic (proportional to its correlation with the causal variant) than a 'non-causal' variant with no such correlation. Therefore, on average, variants with higher LD Scores (more correlations) are more likely to be correlated with a true causal variants. As such, when genome-wide association test statistics (χ²) are regressed on LD scores, the slope provides an estimate of the heritability that can be explained by common genetic variants (*observed h^2^_SNP_)*. However, estimating variance components assumes that traits are continuous, whereas our treatment phenotypes are binary. We assume a liability-threshold model, meaning that the binary phenotype results from splitting the sample at an unknown threshold on an underlying continuous distribution of liability [(Falconer 1965)](https://paperpile.com/c/ir95Fc/iu0OV). Splitting the underlying distribution in this way results in an under-estimation of heritability [(Visscher, Hill, and Wray 2008)](https://paperpile.com/c/ir95Fc/LUhZp). Therefore, observed heritability estimates were transformed to the liability scale using LDSC. This transformation requires making assumptions about the proportion of cases and controls for each trait present in the population, which is difficult to do with treatment-related phenotypes. Therefore, we estimate *h^2^_SNP_* on the liability scale at the full range of population prevalence estimates (figure 1b). Genetic correlations for pairs of phenotypes were estimated by taking the product of the association z-scores at each genetic variant for each phenotype and regressing on the LD score for that variant (using LDSC;[(Bulik-Sullivan et al. 2015)](https://paperpile.com/c/ir95Fc/H6Yc).

# Comparing heritability estimates and genetic correlations using block jackknife and LD Score

We computed the heritability of treatment-related behaviours and genetic correlations using LD score regression [(Bulik-Sullivan et al. 2015)](https://paperpile.com/c/ir95Fc/H6Yc). The same software can be used to determine whether a pair of heritability estimates, or a pair of genetic correlations are significantly different, using a block jackknife approach. For example, define four phenotypes: A, B, C, and D. Global estimates of heritability denoted *H(A)*, *H(B)*, *H(C)* and *H(D)* and global estimates these genetic correlations, denoted *r(A,B)* and *r(C,D)*, can be computed using LD Score. The same software can output jackknife delete values for genetic covariance: *G(A,B)*, *G(C,D)*, as well as for heritability: *H(A,B)* and *H(C,D)*. These jackknife delete values are estimated by excluding blocks of values (here, number of blocks n = 200). The n-dimensional vectors *G(A,B)*, *G(C,D)*, *H(A,B)* and *H(C,D)* can be used to generate genetic correlation delete values *R(A,B)* and *R(C,D)*. The difference between the global estimates *r(A,B)* and *r(C,D)* is *d(AB,CD)* and the difference between the vectors *R(A,B)* and *R(C,D)* is *D(AB,CD)*. The global genetic correlation difference *d(AB,CD)* and the delete values *D(AB,CD)* are used to compute jackknife pseudovalues.

The ith pseudovalue is: $P_{i}(AB,CD) = n \times d (AB,CD) -(n-1)*D_{i}(AB,CD)$

The mean and variance of the jackknife pseudovalues are:

$$m(AB,CD) = \frac{1}{n - 1}\sum_{i=1}^{n} (P_{i}(AB,CD)$$

$$v(AB,CD) = \frac{1}{n - 1}\sum_{i=1}^{n} (P_{i}(AB,CD) - m(AB,CD))^{2}$$

The jackknife estimate of the difference between the two correlations m(AB,CD) can then be compared to test H0 : θ = θ0 (where θ0 = 0 for no difference between genetic correlations), and a p-value can be derived from the z statistic:

$$z(AB,CD) = \frac{m(AB,CD) - \Theta_{0}}{\sqrt{(1/n) \times v(AB,CD)}}$$

# Full list of GWAS traits used for genetic correlations (with reference)

Major depressive disorder [(Wray et al. 2018)](https://paperpile.com/c/ir95Fc/c8u3s)
Anxiety disorders [(Purves et al. 2017)](https://paperpile.com/c/ir95Fc/GcAbC)
Bipolar disorder [(Psychiatric GWAS Consortium Bipolar Disorder Working Group 2011)](https://paperpile.com/c/ir95Fc/LQH7v)
Schizophrenia [(Ripke et al. 2014)](https://paperpile.com/c/ir95Fc/Z9Ctl)
Autism Spectrum Disorder [(Autism Spectrum Disorders Working Group of The Psychiatric Genomics Consortium 2017)](https://paperpile.com/c/ir95Fc/Ro0Ns)
ADHD [(Demontis et al. 2018)](https://paperpile.com/c/ir95Fc/UhH4a)
Anorexia nervosa [(Bulik et al. 2017)](https://paperpile.com/c/ir95Fc/XUEkd)
Borderline personality disorder [(Witt et al. 2017)](https://paperpile.com/c/ir95Fc/kwhDU)
Cross-disorder psychopathology [(Cross-Disorder Group of the Psychiatric Genomics Consortium 2013)](https://paperpile.com/c/ir95Fc/0H7B8)
Alcohol dependence [(Walters et al. 2018)](https://paperpile.com/c/ir95Fc/aauI3)
Depressive symptoms ([(Okbay et al. 2016)](https://paperpile.com/c/ir95Fc/y2cFr)
Insomnia [(Hammerschlag et al. 2017)](https://paperpile.com/c/ir95Fc/n9wxM)
Tiredness [(Deary et al. 2018)](https://paperpile.com/c/ir95Fc/SPwbE)
Migraine [(Gormley et al. 2016)](https://paperpile.com/c/ir95Fc/RD9TI)
BMI [(Yengo et al. 2018)](https://paperpile.com/c/ir95Fc/og6jh)
Neuroticism and subjective well-being [(Okbay et al. 2016)](https://paperpile.com/c/ir95Fc/y2cFr)
Extraversion, Openness to experience, and Conscientiousness [(de Moor et al. 2010)](https://paperpile.com/c/ir95Fc/82zqH)
Adult IQ [(Savage et al. 2018)](https://paperpile.com/c/ir95Fc/p2AYO)
Child IQ [(Benyamin et al. 2014)](https://paperpile.com/c/ir95Fc/O6VvK)
Educational attainment [(Lee et al. 2018)](https://paperpile.com/c/ir95Fc/4bpwk)
Social deprivation [(Hill et al. 2016)](https://paperpile.com/c/ir95Fc/Zqlxp)
Alcohol use [(Schumann et al. 2016)](https://paperpile.com/c/ir95Fc/UcTYP)
Cannabis use [(Stringer et al. 2016)](https://paperpile.com/c/ir95Fc/jctJU)

Supplementary Results

# LD score regression SNP heritability and intercept estimates

## Supplementary Table 1. LD score regression SNP heritability and intercept estimates, and standard errors from analyses in the full sample and from stratified analyses

|  |  | Treatment-seeking | | Treatment-receipt | | Self-help | | Self-medication | |
| --- | --- | --- | --- | --- | --- | --- | --- | --- | --- |
| Full-sample | SNP h2 (se) | 0.0457 | (0.0071) | -0.0067 | (0.0089) | 0.0232 | (0.0061) | 0.034 | (0.0069) |
|  | Intercept (se) | 1.0097 | (0.0058) | 1.0036 | (0.0058) | 1.0039 | (0.0063) | 0.9953 | (0.0071) |
| Cases | SNP h2 (se) | 0.0335 | (0.0156) | -0.017 | (0.0157) | 0.0309 | (0.0143) | 0.0258 | (0.0153) |
|  | Intercept (se) | 0.9985 | (0.0063) | 1.0061 | (0.0058) | 0.9961 | (0.0061) | 0.9991 | (0.0069) |
| Controls | SNP h2 (se) | 0.0491 | (0.0153) | 0.0281 | (0.0287) | 0.0075 | (0.0124) | 0.0274 | (0.0168) |
|  | Intercept (se) | 1.0047 | (0.0065) | 0.9936 | (0.0059) | 1.002 | (0.0058) | 0.994 | (0.0068) |
| Males | SNP h2 (se) | 0.0601 | (0.0169) | -0.0113 | (0.0292) | -0.0046 | (0.016) | 0.0484 | (0.0184) |
|  | Intercept (se) | 0.9946 | (0.0054) | 1.0038 | (0.006) | 1.0037 | (0.006) | 0.9964 | (0.0067) |
| Females | SNP h2 (se) | 0.0381 | (0.0097) | 0.01 | (0.0131) | 0.0236 | (0.0092) | 0.027 | (0.0094) |
|  | Intercept (se) | 1.0082 | (0.0061) | 0.9968 | (0.0058) | 1.0021 | (0.006) | 1.0003 | (0.0064) |

# Manhattan and QQ plots of genome-wide associtation p-values, for analyses in the full sample

## Supplementary Figure 1. Manhattan and QQ plots for GWAS of treatment-seeking in the full sample


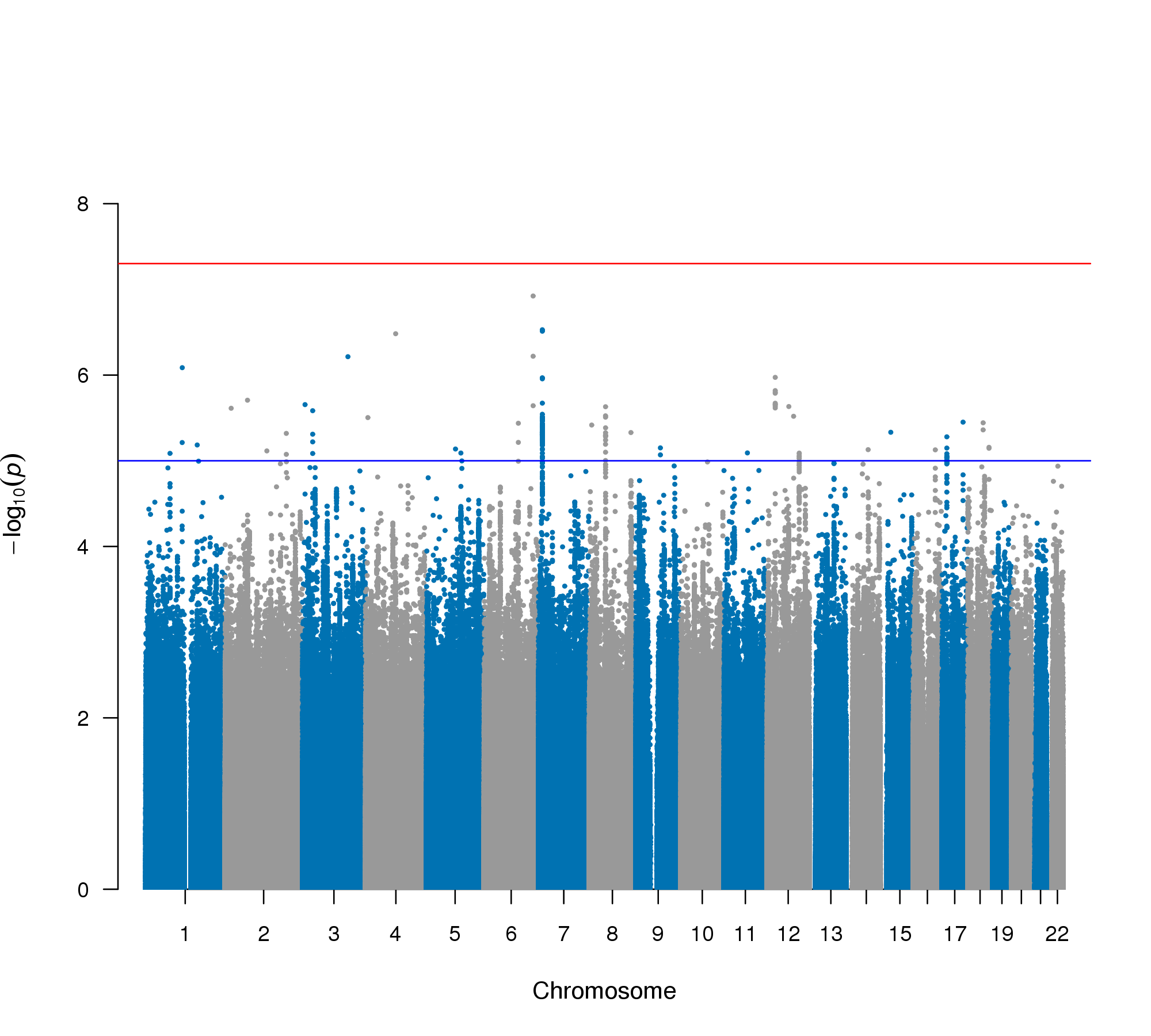

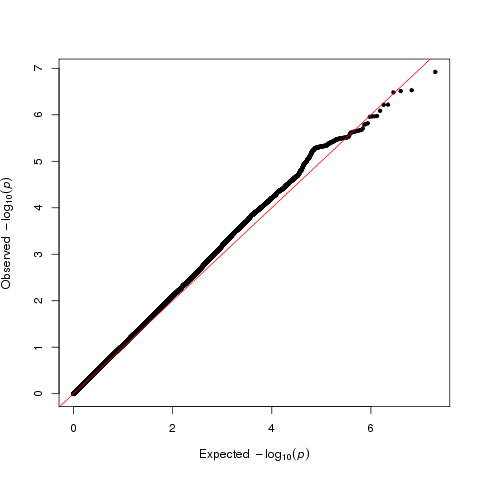


## Supplementary Figure 2. Manhattan and QQ plots for GWAS of treatment-receipt in the full sample


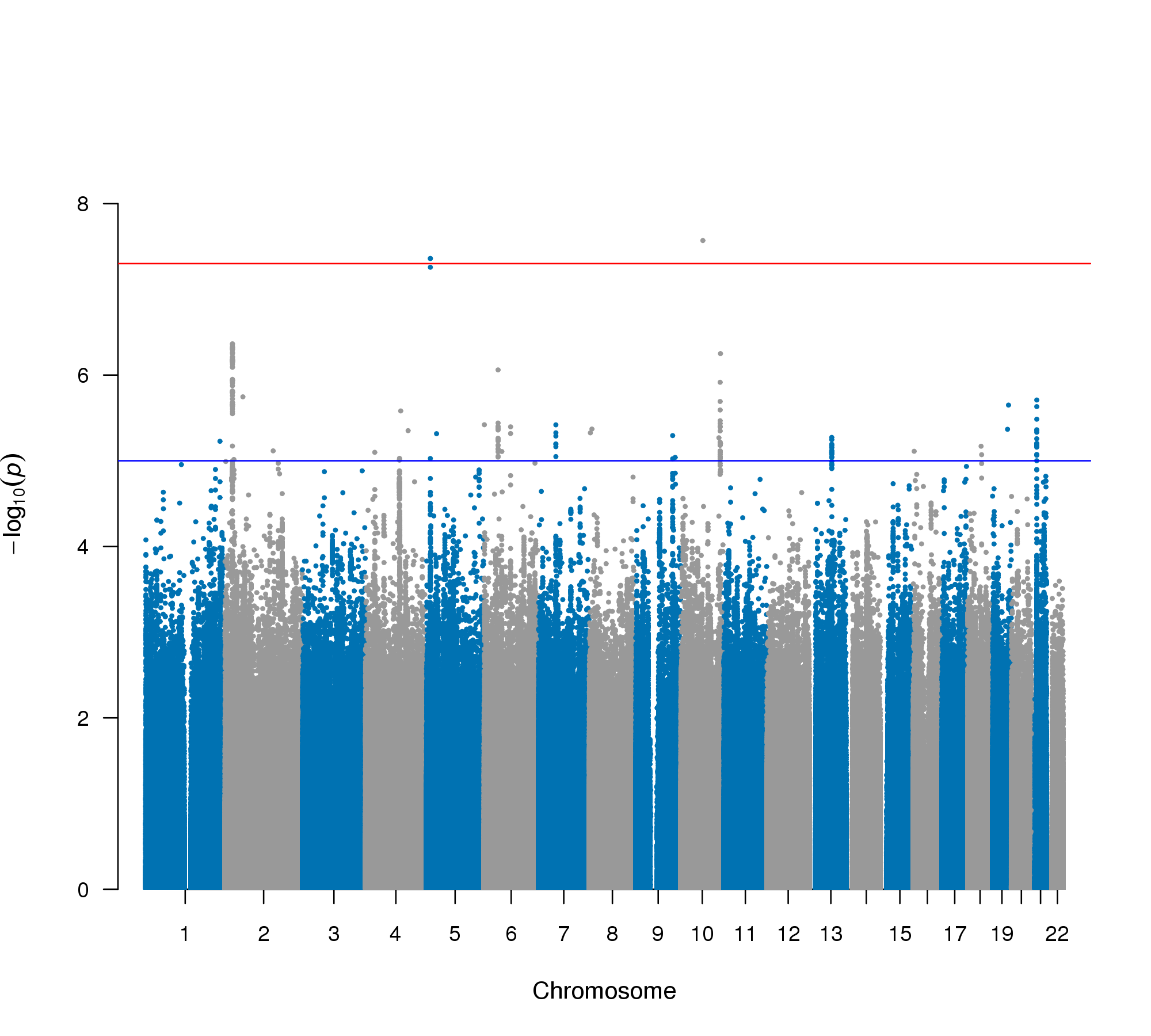

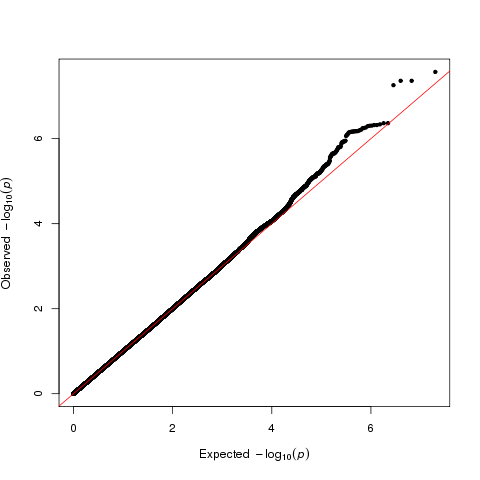


## Supplementary Figure 3. Manhattan and QQ plots for GWAS of Self-medication in the full sample


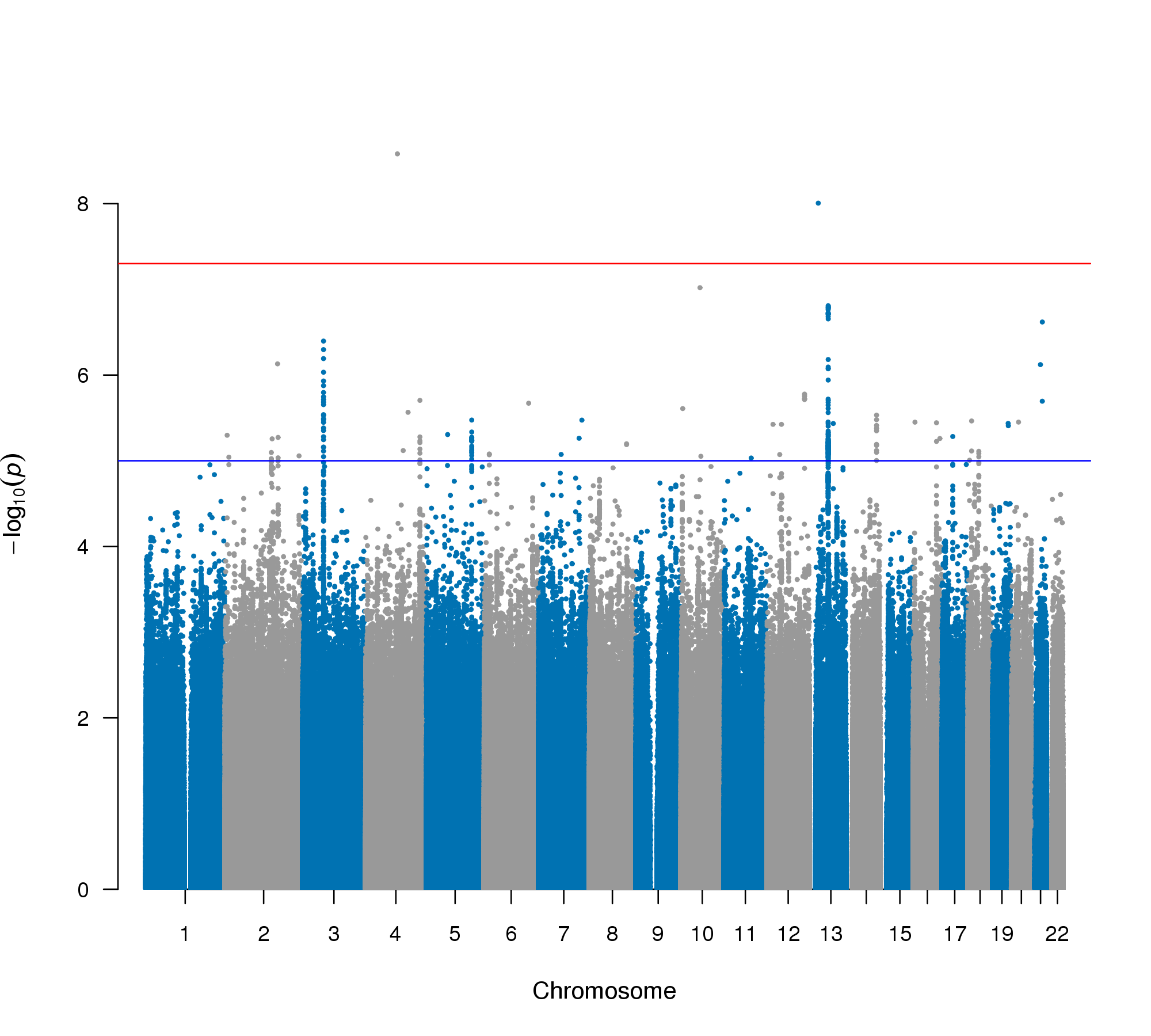

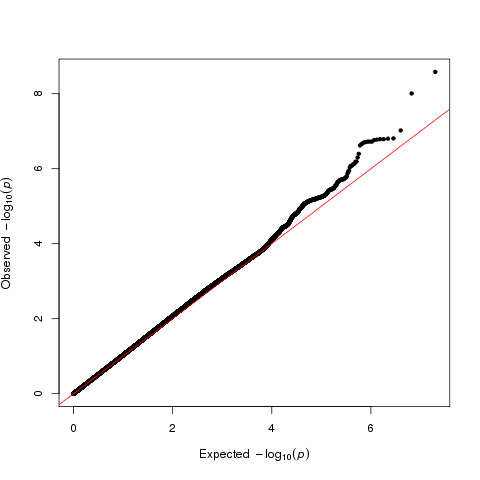


##

## Supplementary Figure 4. Manhattan and QQ plots for GWAS of Self-help in the full sample


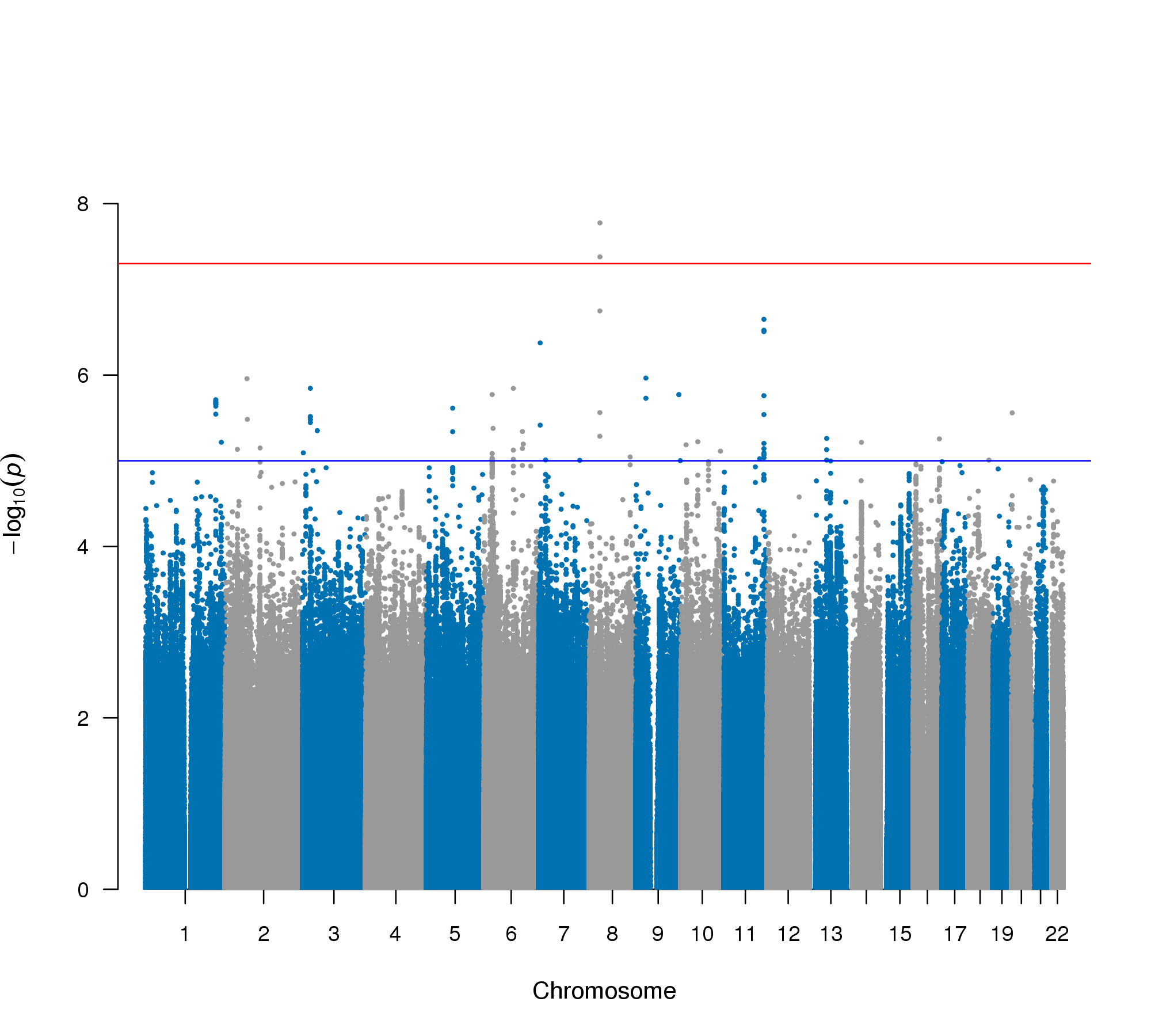

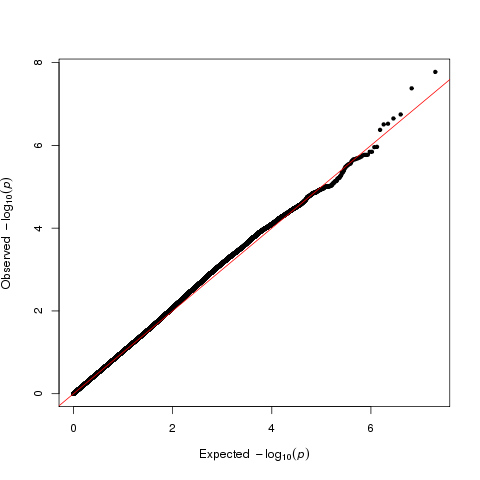


# Genetic correlations, stratified by case-status and sex

## Supplementary Figure 5. Genetic correlations of treatment phenotypes and external traits in the “case/control” stratified samples


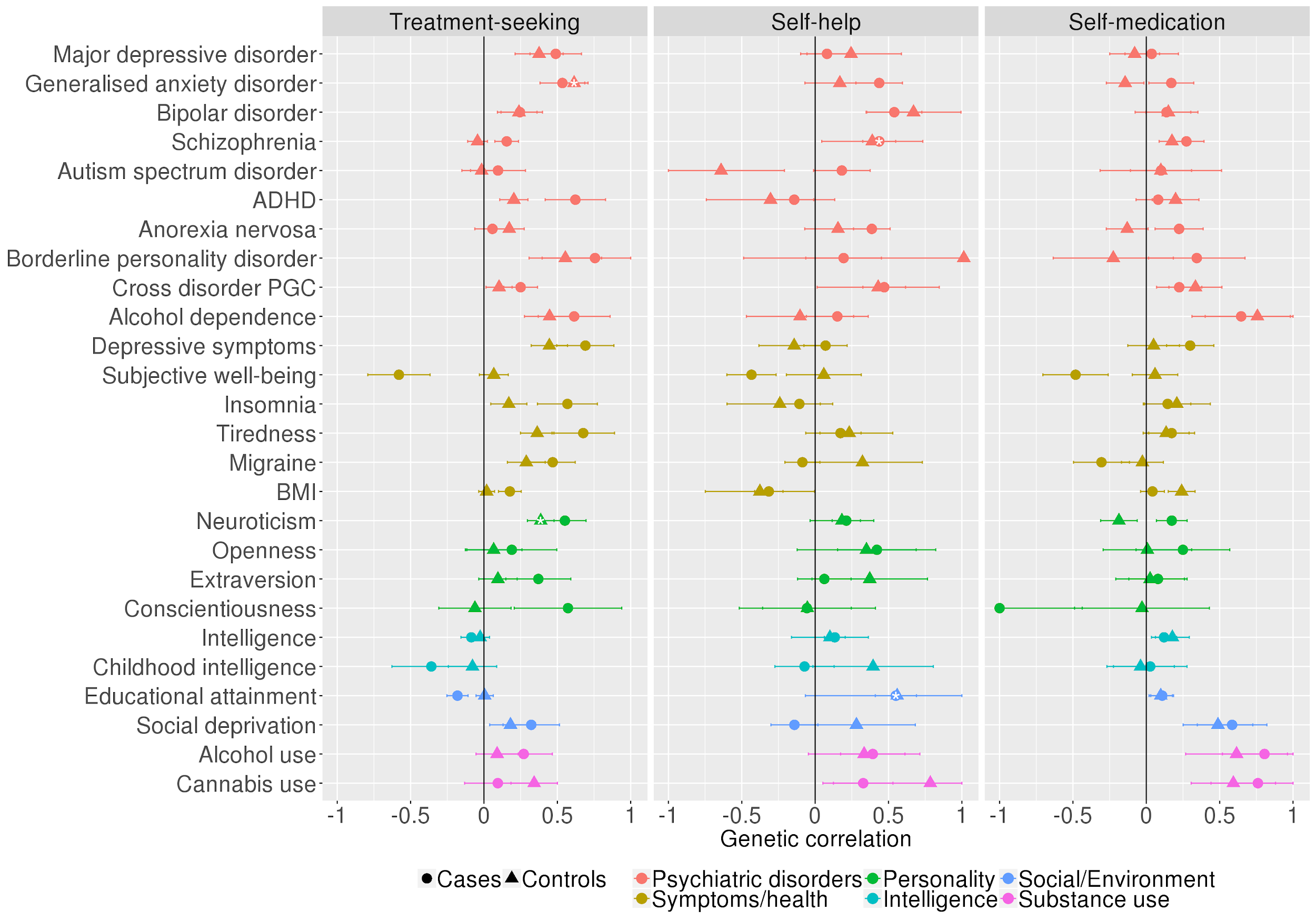


## Supplementary Figure 6. Genetic correlations of treatment phenotypes and external traits in the sex stratified samples


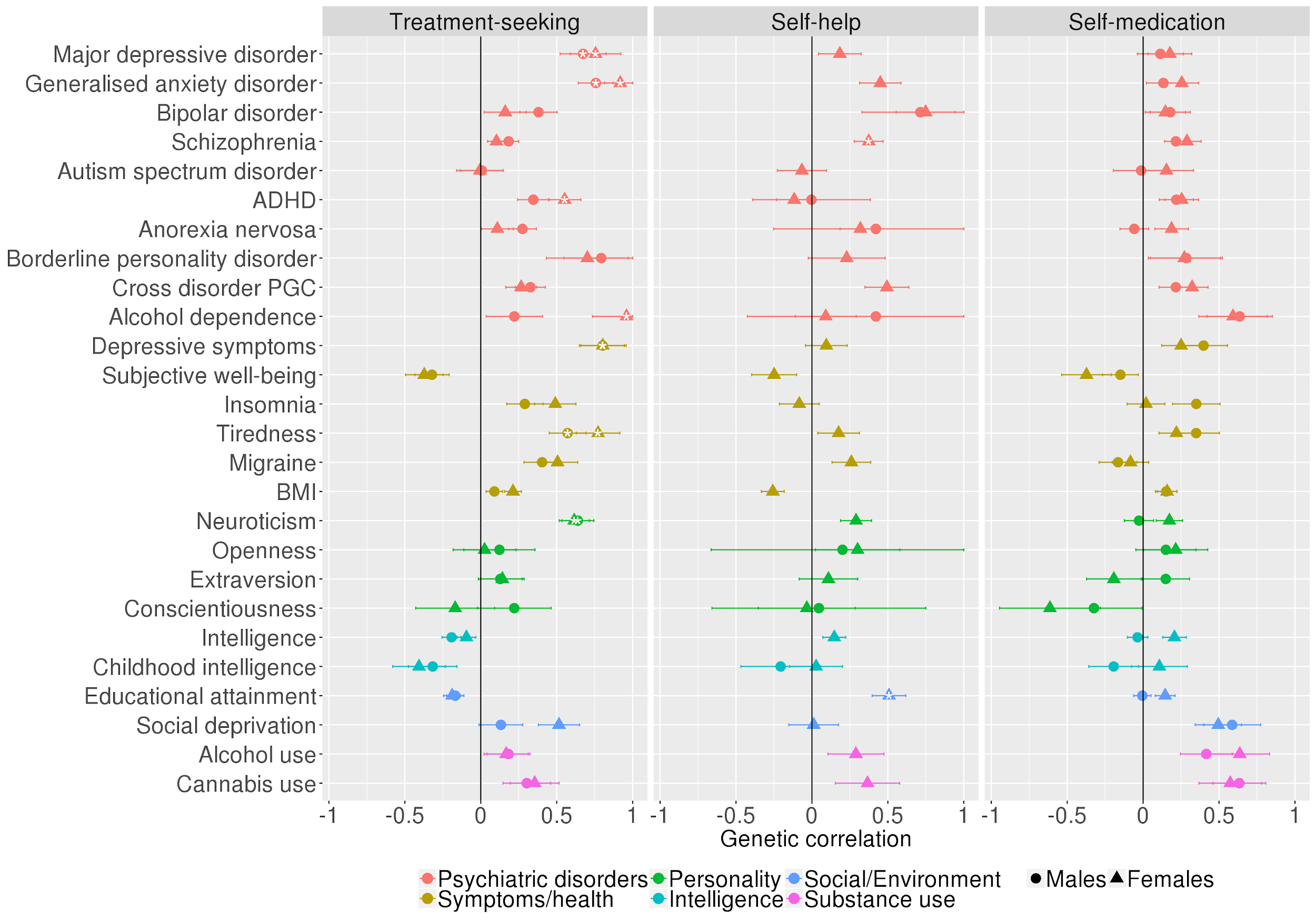


##

## Supplementary table 2. Genetic correlations between treatment phenotypes and external traits (psychiatric disorders, behavioural traits; ordered by significance)

| **External trait** | **Treatment phenotype** | **Sample** | **rg** | **se** | **z** | **p** |
| --- | --- | --- | --- | --- | --- | --- |
| Neuroticism | Formal treatment-seeking | Full | 0.67 | 0.02 | 34.11 | 5.82E-255 |
| Generalised anxiety disorder | Formal treatment-seeking | Full | 0.90 | 0.03 | 27.49 | 2.31E-166 |
| Neuroticism | Formal treatment-seeking | Females | 0.64 | 0.03 | 25.32 | 1.76E-141 |
| Depressive symptoms | Formal treatment-seeking | Full | 0.85 | 0.03 | 25.05 | 1.64E-138 |
| Neuroticism | Formal treatment-seeking | Males | 0.70 | 0.03 | 23.06 | 1.25E-117 |
| Generalised anxiety disorder | Formal treatment-seeking | Females | 0.86 | 0.04 | 22.68 | 6.87E-114 |
| Depressive symptoms | Formal treatment-seeking | Females | 0.83 | 0.04 | 20.23 | 5.33E-91 |
| Generalised anxiety disorder | Formal treatment-seeking | Males | 0.90 | 0.05 | 18.70 | 5.29E-78 |
| Tiredness | Formal treatment-seeking | Full | 0.66 | 0.04 | 17.29 | 5.53E-67 |
| Major depressive disorder | Formal treatment-seeking | Full | 0.86 | 0.05 | 16.00 | 1.31E-57 |
| Subjective well being | Formal treatment-seeking | Full | -0.61 | 0.04 | -15.73 | 9.30E-56 |
| Generalised anxiety disorder | Broad treatment-seeking | Full | 0.85 | 0.05 | 15.46 | 6.49E-54 |
| Depressive symptoms | Formal treatment-seeking | Males | 0.82 | 0.05 | 15.27 | 1.15E-52 |
| Schizophrenia | Formal treatment-seeking | Full | 0.36 | 0.02 | 15.19 | 3.90E-52 |
| Tiredness | Formal treatment-seeking | Females | 0.65 | 0.04 | 14.97 | 1.16E-50 |
| Cross disorder PGC | Formal treatment-seeking | Full | 0.51 | 0.03 | 14.86 | 5.66E-50 |
| Major depressive disorder | Formal treatment-seeking | Females | 0.84 | 0.06 | 13.56 | 6.77E-42 |
| ADHD | Formal treatment-seeking | Full | 0.41 | 0.03 | 12.58 | 2.76E-36 |
| ADHD | Formal treatment-seeking | Females | 0.48 | 0.04 | 12.43 | 1.82E-35 |
| Major depressive disorder | Formal treatment-seeking | Males | 0.84 | 0.07 | 12.42 | 2.06E-35 |
| Subjective well being | Formal treatment-seeking | Females | -0.56 | 0.05 | -12.40 | 2.48E-35 |
| Schizophrenia | Formal treatment-seeking | Males | 0.42 | 0.04 | 12.09 | 1.20E-33 |
| Tiredness | Formal treatment-seeking | Males | 0.64 | 0.05 | 11.71 | 1.11E-31 |
| Subjective well being | Formal treatment-seeking | Males | -0.64 | 0.06 | -11.62 | 3.34E-31 |
| Cross disorder PGC | Formal treatment-seeking | Males | 0.54 | 0.05 | 11.47 | 1.97E-30 |
| Educational attainment | Formal treatment-seeking | Females | -0.27 | 0.02 | -11.14 | 8.21E-29 |
| Educational attainment | Formal treatment-seeking | Full | -0.22 | 0.02 | -11.03 | 2.60E-28 |
| Cross disorder PGC | Formal treatment-seeking | Females | 0.45 | 0.04 | 11.01 | 3.25E-28 |
| Schizophrenia | Formal treatment-seeking | Females | 0.29 | 0.03 | 10.44 | 1.62E-25 |
| Insomnia | Formal treatment-seeking | Full | 0.44 | 0.04 | 9.74 | 2.05E-22 |
| Neuroticism | Broad treatment-seeking | Full | 0.62 | 0.07 | 9.41 | 4.80E-21 |
| Generalised anxiety disorder | Broad treatment-seeking | Females | 0.92 | 0.10 | 8.89 | 6.37E-19 |
| Depressive symptoms | Broad treatment-seeking | Full | 0.80 | 0.09 | 8.77 | 1.79E-18 |
| Insomnia | Formal treatment-seeking | Females | 0.46 | 0.05 | 8.76 | 2.03E-18 |
| Intelligence | Formal treatment-seeking | Females | -0.21 | 0.02 | -8.68 | 3.97E-18 |
| Bipolar disorder | Formal treatment-seeking | Full | 0.35 | 0.04 | 8.46 | 2.75E-17 |
| Migraine | Formal treatment-seeking | Full | 0.32 | 0.04 | 8.40 | 4.40E-17 |
| Tiredness | Broad treatment-seeking | Full | 0.68 | 0.08 | 8.09 | 5.86E-16 |
| Social deprivation | Formal treatment-seeking | Full | 0.44 | 0.06 | 7.86 | 3.80E-15 |
| Intelligence | Formal treatment-seeking | Full | -0.17 | 0.02 | -7.83 | 4.86E-15 |
| Migraine | Formal treatment-seeking | Females | 0.33 | 0.04 | 7.71 | 1.31E-14 |
| BMI | Formal treatment-seeking | Females | 0.19 | 0.03 | 7.69 | 1.47E-14 |
| Alcohol dependence | Formal treatment-seeking | Full | 0.57 | 0.08 | 7.40 | 1.32E-13 |
| Alcohol dependence | Formal treatment-seeking | Females | 0.58 | 0.08 | 6.85 | 7.29E-12 |
| ADHD | Broad treatment-seeking | Full | 0.47 | 0.07 | 6.78 | 1.23E-11 |
| Social deprivation | Formal treatment-seeking | Females | 0.40 | 0.06 | 6.66 | 2.76E-11 |
| Generalised anxiety disorder | Broad treatment-seeking | Males | 0.76 | 0.11 | 6.63 | 3.30E-11 |
| Bipolar disorder | Formal treatment-seeking | Females | 0.30 | 0.05 | 6.63 | 3.32E-11 |
| Major depressive disorder | Broad treatment-seeking | Full | 0.72 | 0.11 | 6.63 | 3.45E-11 |
| Insomnia | Formal treatment-seeking | Males | 0.39 | 0.06 | 6.53 | 6.39E-11 |
| Bipolar disorder | Formal treatment-seeking | Males | 0.37 | 0.06 | 6.50 | 7.82E-11 |
| Generalised anxiety disorder | Broad treatment-seeking | Controls | 0.61 | 0.09 | 6.49 | 8.79E-11 |
| Anorexia nervosa | Formal treatment-seeking | Full | 0.22 | 0.04 | 6.25 | 4.09E-10 |
| Neuroticism | Broad treatment-seeking | Females | 0.62 | 0.10 | 6.22 | 5.11E-10 |
| Educational attainment | Self-help | Full | 0.43 | 0.07 | 6.15 | 7.76E-10 |
| Neuroticism | Broad treatment-seeking | Males | 0.64 | 0.11 | 6.08 | 1.19E-09 |
| Social deprivation | Formal treatment-seeking | Males | 0.46 | 0.08 | 6.05 | 1.42E-09 |
| Schizophrenia | Self-help | Full | 0.41 | 0.07 | 5.95 | 2.72E-09 |
| ADHD | Formal treatment-seeking | Males | 0.29 | 0.05 | 5.93 | 2.96E-09 |
| Bipolar disorder | Self-help | Full | 0.64 | 0.11 | 5.72 | 1.06E-08 |
| BMI | Formal treatment-seeking | Full | 0.13 | 0.02 | 5.70 | 1.22E-08 |
| Depressive symptoms | Broad treatment-seeking | Males | 0.80 | 0.14 | 5.62 | 1.95E-08 |
| Anorexia nervosa | Formal treatment-seeking | Males | 0.27 | 0.05 | 5.50 | 3.80E-08 |
| Educational attainment | Formal treatment-seeking | Males | -0.14 | 0.03 | -5.42 | 5.81E-08 |
| Tiredness | Broad treatment-seeking | Females | 0.77 | 0.14 | 5.39 | 7.09E-08 |
| Migraine | Formal treatment-seeking | Males | 0.26 | 0.05 | 5.24 | 1.61E-07 |
| Alcohol dependence | Formal treatment-seeking | Males | 0.50 | 0.10 | 5.21 | 1.84E-07 |
| Migraine | Broad treatment-seeking | Full | 0.46 | 0.09 | 5.21 | 1.85E-07 |
| Depressive symptoms | Broad treatment-seeking | Females | 0.80 | 0.15 | 5.20 | 1.98E-07 |
| ADHD | Broad treatment-seeking | Females | 0.55 | 0.11 | 5.17 | 2.31E-07 |
| Cross disorder PGC | Self-help | Full | 0.52 | 0.10 | 5.06 | 4.27E-07 |
| Borderline personality disorder | Formal treatment-seeking | Full | 0.57 | 0.11 | 5.04 | 4.59E-07 |
| Generalised anxiety disorder | Self-help | Full | 0.51 | 0.10 | 4.89 | 1.01E-06 |
| Social deprivation | Self-medication | Full | 0.55 | 0.11 | 4.80 | 1.62E-06 |
| Anorexia nervosa | Formal treatment-seeking | Females | 0.18 | 0.04 | 4.73 | 2.23E-06 |
| Tiredness | Broad treatment-seeking | Males | 0.57 | 0.12 | 4.73 | 2.28E-06 |
| Educational attainment | Self-help | Females | 0.51 | 0.11 | 4.59 | 4.34E-06 |
| Cannabis use | Self-medication | Full | 0.60 | 0.13 | 4.57 | 4.86E-06 |
| Major depressive disorder | Broad treatment-seeking | Females | 0.76 | 0.17 | 4.55 | 5.36E-06 |
| Alcohol dependence | Broad treatment-seeking | Full | 0.65 | 0.14 | 4.53 | 5.82E-06 |
| Borderline personality disorder | Formal treatment-seeking | Females | 0.56 | 0.13 | 4.47 | 7.85E-06 |
| Major depressive disorder | Broad treatment-seeking | Males | 0.67 | 0.15 | 4.46 | 8.24E-06 |
| Schizophrenia | Self-medication | Full | 0.26 | 0.06 | 4.41 | 1.05E-05 |
| Alcohol use | Self-medication | Full | 0.53 | 0.12 | 4.40 | 1.10E-05 |
| Subjective well being | Broad treatment-seeking | Full | -0.35 | 0.08 | -4.34 | 1.40E-05 |
| Alcohol dependence | Self-medication | Full | 0.62 | 0.14 | 4.31 | 1.66E-05 |
| Alcohol dependence | Broad treatment-seeking | Females | 0.96 | 0.22 | 4.28 | 1.89E-05 |
| Neuroticism | Broad treatment-seeking | Controls | 0.39 | 0.09 | 4.27 | 1.95E-05 |
| Borderline personality disorder | Formal treatment-seeking | Males | 0.53 | 0.13 | 4.24 | 2.25E-05 |
| Educational attainment | Broad treatment-seeking | Full | -0.17 | 0.04 | -4.20 | 2.63E-05 |
| Insomnia | Broad treatment-seeking | Full | 0.40 | 0.10 | 4.19 | 2.75E-05 |
| Cross disorder PGC | Broad treatment-seeking | Full | 0.30 | 0.07 | 4.18 | 2.87E-05 |
| BMI | Self-help | Full | -0.24 | 0.06 | -4.05 | 5.04E-05 |
| BMI | Broad treatment-seeking | Full | 0.16 | 0.04 | 4.05 | 5.11E-05 |
| Cannabis use | Formal treatment-seeking | Full | 0.26 | 0.06 | 4.05 | 5.11E-05 |
| Schizophrenia | Self-help | Females | 0.37 | 0.10 | 3.94 | 8.26E-05 |
| Educational attainment | Self-help | Cases | 0.55 | 0.14 | 3.92 | 8.82E-05 |
| Schizophrenia | Self-help | Cases | 0.44 | 0.11 | 3.89 | 9.90E-05 |
| Bipolar disorder | Self-help | Females | 0.75 | 0.19 | 3.86 | 0.0001 |
| BMI | Broad treatment-seeking | Females | 0.21 | 0.06 | 3.80 | 0.0001 |
| Migraine | Broad treatment-seeking | Females | 0.51 | 0.13 | 3.81 | 0.0001 |
| Social deprivation | Broad treatment-seeking | Females | 0.52 | 0.14 | 3.81 | 0.0001 |
| Neuroticism | Broad treatment-seeking | Cases | 0.55 | 0.14 | 3.83 | 0.0001 |
| Cross disorder PGC | Self-medication | Full | 0.28 | 0.08 | 3.73 | 0.0002 |
| Cannabis use | Formal treatment-seeking | Females | 0.26 | 0.07 | 3.75 | 0.0002 |
| Neuroticism | Self-help | Full | 0.28 | 0.08 | 3.61 | 0.0003 |
| Cannabis use | Self-medication | Males | 0.63 | 0.17 | 3.63 | 0.0003 |
| Borderline personality disorder | Broad treatment-seeking | Full | 0.75 | 0.21 | 3.61 | 0.0003 |
| Insomnia | Broad treatment-seeking | Females | 0.49 | 0.14 | 3.61 | 0.0003 |
| Depressive symptoms | Broad treatment-seeking | Controls | 0.45 | 0.12 | 3.61 | 0.0003 |
| Intelligence | Formal treatment-seeking | Males | -0.11 | 0.03 | -3.64 | 0.0003 |
| Depressive symptoms | Broad treatment-seeking | Cases | 0.69 | 0.19 | 3.55 | 0.0004 |
| Social deprivation | Broad treatment-seeking | Full | 0.35 | 0.10 | 3.46 | 0.0005 |
| Generalised anxiety disorder | Broad treatment-seeking | Cases | 0.53 | 0.15 | 3.51 | 0.0005 |
| BMI | Self-help | Females | -0.26 | 0.08 | -3.42 | 0.0006 |
| Cross disorder PGC | Self-help | Females | 0.49 | 0.14 | 3.42 | 0.0006 |
| Migraine | Broad treatment-seeking | Males | 0.40 | 0.12 | 3.38 | 0.0007 |
| ADHD | Broad treatment-seeking | Males | 0.35 | 0.10 | 3.34 | 0.0008 |
| Educational attainment | Broad treatment-seeking | Females | -0.19 | 0.06 | -3.34 | 0.0008 |
| Cross disorder PGC | Broad treatment-seeking | Males | 0.33 | 0.10 | 3.33 | 0.0009 |
| Generalised anxiety disorder | Self-help | Females | 0.45 | 0.14 | 3.30 | 0.001 |
| BMI | Self-help | Cases | -0.32 | 0.10 | -3.27 | 0.0011 |
| Alcohol use | Self-medication | Females | 0.64 | 0.20 | 3.26 | 0.0011 |
| Cross disorder PGC | Self-help | Cases | 0.47 | 0.15 | 3.24 | 0.0012 |
| Social deprivation | Self-medication | Females | 0.50 | 0.15 | 3.24 | 0.0012 |
| Borderline personality disorder | Broad treatment-seeking | Males | 0.79 | 0.25 | 3.23 | 0.0012 |
| Tiredness | Broad treatment-seeking | Controls | 0.36 | 0.11 | 3.19 | 0.0014 |
| Tiredness | Broad treatment-seeking | Cases | 0.68 | 0.21 | 3.17 | 0.0015 |
| Anorexia nervosa | Self-help | Full | 0.27 | 0.09 | 3.16 | 0.0016 |
| Social deprivation | Self-medication | Males | 0.59 | 0.19 | 3.13 | 0.0017 |
| BMI | Self-medication | Full | 0.16 | 0.05 | 3.14 | 0.0017 |
| Anorexia nervosa | Self-help | Cases | 0.39 | 0.12 | 3.10 | 0.0019 |
| Schizophrenia | Self-medication | Females | 0.29 | 0.09 | 3.10 | 0.0019 |
| Bipolar disorder | Broad treatment-seeking | Males | 0.38 | 0.12 | 3.11 | 0.0019 |
| Intelligence | Broad treatment-seeking | Males | -0.19 | 0.06 | -3.10 | 0.0019 |
| Depressive symptoms | Self-medication | Full | 0.32 | 0.10 | 3.09 | 0.002 |
| Cross disorder PGC | Self-medication | Females | 0.32 | 0.10 | 3.08 | 0.0021 |
| Migraine | Broad treatment-seeking | Cases | 0.47 | 0.15 | 3.05 | 0.0023 |
| Tiredness | Self-medication | Full | 0.28 | 0.09 | 3.04 | 0.0024 |
| Schizophrenia | Broad treatment-seeking | Full | 0.14 | 0.05 | 3.02 | 0.0025 |
| ADHD | Broad treatment-seeking | Cases | 0.62 | 0.21 | 3.03 | 0.0025 |
| Anorexia nervosa | Broad treatment-seeking | Males | 0.27 | 0.09 | 3.02 | 0.0026 |
| Subjective well being | Broad treatment-seeking | Females | -0.37 | 0.12 | -3.01 | 0.0026 |
| Intelligence | Broad treatment-seeking | Full | -0.13 | 0.05 | -2.96 | 0.003 |
| Educational attainment | Broad treatment-seeking | Males | -0.17 | 0.06 | -2.96 | 0.0031 |
| Alcohol dependence | Self-medication | Males | 0.64 | 0.22 | 2.95 | 0.0032 |
| Cannabis use | Formal treatment-seeking | Males | 0.25 | 0.08 | 2.94 | 0.0033 |
| ADHD | Self-medication | Full | 0.22 | 0.08 | 2.92 | 0.0035 |
| Schizophrenia | Self-medication | Males | 0.22 | 0.07 | 2.88 | 0.0039 |
| Bipolar disorder | Self-help | Cases | 0.54 | 0.19 | 2.87 | 0.0041 |
| Cannabis use | Broad treatment-seeking | Full | 0.34 | 0.12 | 2.85 | 0.0043 |
| Childhood intelligence | Broad treatment-seeking | Full | -0.37 | 0.13 | -2.85 | 0.0043 |
| Neuroticism | Self-help | Females | 0.29 | 0.10 | 2.84 | 0.0044 |
| Subjective well being | Broad treatment-seeking | Males | -0.32 | 0.11 | -2.85 | 0.0044 |
| Alcohol use | Self-medication | Cases | 0.81 | 0.29 | 2.81 | 0.005 |
| Insomnia | Broad treatment-seeking | Cases | 0.57 | 0.20 | 2.79 | 0.0053 |
| Cannabis use | Self-medication | Females | 0.57 | 0.21 | 2.78 | 0.0054 |
| Major depressive disorder | Broad treatment-seeking | Cases | 0.49 | 0.18 | 2.79 | 0.0054 |
| Childhood intelligence | Formal treatment-seeking | Females | -0.21 | 0.07 | -2.78 | 0.0054 |
| Cannabis use | Self-help | Full | 0.44 | 0.16 | 2.77 | 0.0055 |
| Subjective well being | Self-help | Full | -0.31 | 0.11 | -2.76 | 0.0057 |
| Schizophrenia | Broad treatment-seeking | Males | 0.18 | 0.07 | 2.77 | 0.0057 |
| Generalised anxiety disorder | Self-help | Cases | 0.44 | 0.16 | 2.75 | 0.0059 |
| Bipolar disorder | Broad treatment-seeking | Full | 0.26 | 0.10 | 2.75 | 0.006 |
| Subjective well being | Broad treatment-seeking | Cases | -0.58 | 0.21 | -2.73 | 0.0064 |
| Intelligence | Self-medication | Females | 0.21 | 0.08 | 2.65 | 0.008 |
| Alcohol dependence | Self-medication | Females | 0.59 | 0.22 | 2.64 | 0.0084 |
| BMI | Self-medication | Controls | 0.24 | 0.09 | 2.63 | 0.0085 |
| Cross disorder PGC | Broad treatment-seeking | Females | 0.27 | 0.10 | 2.62 | 0.0089 |
| Alcohol dependence | Broad treatment-seeking | Controls | 0.45 | 0.17 | 2.62 | 0.0089 |
| Borderline personality disorder | Broad treatment-seeking | Females | 0.70 | 0.27 | 2.61 | 0.0091 |
| Anorexia nervosa | Broad treatment-seeking | Full | 0.19 | 0.07 | 2.59 | 0.0096 |
| Childhood intelligence | Formal treatment-seeking | Full | -0.18 | 0.07 | -2.59 | 0.0097 |
| Subjective well being | Self-help | Cases | -0.43 | 0.17 | -2.58 | 0.0098 |
| Depressive symptoms | Self-medication | Males | 0.40 | 0.16 | 2.52 | 0.0118 |
| Subjective well being | Self-medication | Full | -0.26 | 0.10 | -2.51 | 0.012 |
| Alcohol dependence | Broad treatment-seeking | Cases | 0.61 | 0.24 | 2.51 | 0.012 |
| Educational attainment | Broad treatment-seeking | Cases | -0.18 | 0.07 | -2.51 | 0.012 |
| BMI | Self-medication | Females | 0.16 | 0.06 | 2.49 | 0.0128 |
| Generalised anxiety disorder | Self-medication | Full | 0.21 | 0.08 | 2.47 | 0.0136 |
| Social deprivation | Self-medication | Cases | 0.58 | 0.24 | 2.47 | 0.0136 |
| Alcohol use | Self-medication | Males | 0.42 | 0.17 | 2.44 | 0.0147 |
| Insomnia | Broad treatment-seeking | Males | 0.29 | 0.12 | 2.43 | 0.0153 |
| Major depressive disorder | Self-help | Full | 0.29 | 0.12 | 2.41 | 0.0161 |
| Anorexia nervosa | Self-help | Females | 0.32 | 0.13 | 2.39 | 0.017 |
| Cannabis use | Self-medication | Cases | 0.76 | 0.32 | 2.38 | 0.0174 |
| Childhood intelligence | Broad treatment-seeking | Females | -0.41 | 0.17 | -2.35 | 0.0186 |
| Tiredness | Self-help | Full | 0.25 | 0.11 | 2.32 | 0.0202 |
| Major depressive disorder | Broad treatment-seeking | Controls | 0.38 | 0.16 | 2.30 | 0.0217 |
| Generalised anxiety disorder | Self-medication | Females | 0.25 | 0.11 | 2.29 | 0.0218 |
| ADHD | Self-medication | Females | 0.25 | 0.11 | 2.29 | 0.022 |
| Schizophrenia | Self-medication | Cases | 0.27 | 0.12 | 2.29 | 0.0221 |
| Tiredness | Self-medication | Males | 0.35 | 0.15 | 2.28 | 0.0225 |
| Subjective well being | Self-medication | Females | -0.37 | 0.16 | -2.28 | 0.0226 |
| BMI | Broad treatment-seeking | Cases | 0.18 | 0.08 | 2.28 | 0.0227 |
| Borderline personality disorder | Broad treatment-seeking | Controls | 0.55 | 0.25 | 2.25 | 0.0247 |
| Insomnia | Self-medication | Males | 0.35 | 0.16 | 2.24 | 0.0248 |
| Migraine | Broad treatment-seeking | Controls | 0.29 | 0.13 | 2.24 | 0.0249 |
| Educational attainment | Self-medication | Females | 0.15 | 0.07 | 2.22 | 0.0261 |
| Cannabis use | Broad treatment-seeking | Females | 0.36 | 0.16 | 2.21 | 0.0269 |
| Neuroticism | Self-help | Cases | 0.21 | 0.10 | 2.20 | 0.0276 |
| Cross disorder PGC | Broad treatment-seeking | Cases | 0.25 | 0.12 | 2.17 | 0.0298 |
| Subjective well being | Self-medication | Cases | -0.48 | 0.22 | -2.17 | 0.0301 |
| Cannabis use | Broad treatment-seeking | Controls | 0.34 | 0.16 | 2.16 | 0.0305 |
| BMI | Self-medication | Males | 0.15 | 0.07 | 2.13 | 0.0334 |
| Alcohol dependence | Self-medication | Controls | 0.76 | 0.36 | 2.13 | 0.0334 |
| ADHD | Broad treatment-seeking | Controls | 0.20 | 0.10 | 2.12 | 0.0337 |
| Borderline personality disorder | Broad treatment-seeking | Cases | 0.76 | 0.36 | 2.10 | 0.0356 |
| Conscientiousness | Formal treatment-seeking | Females | -0.23 | 0.11 | -2.09 | 0.0369 |
| Bipolar disorder | Self-help | Controls | 0.67 | 0.32 | 2.07 | 0.0381 |
| Cannabis use | Self-medication | Controls | 0.59 | 0.29 | 2.06 | 0.0391 |
| Social deprivation | Self-medication | Controls | 0.49 | 0.24 | 2.06 | 0.0396 |
| Migraine | Self-help | Females | 0.26 | 0.13 | 2.04 | 0.0414 |
| Depressive symptoms | Self-help | Full | 0.22 | 0.11 | 2.00 | 0.0452 |
| Schizophrenia | Self-medication | Controls | 0.17 | 0.09 | 2.00 | 0.0456 |
| Neuroticism | Self-medication | Females | 0.17 | 0.09 | 1.99 | 0.0461 |
| Childhood intelligence | Broad treatment-seeking | Males | -0.32 | 0.16 | -1.99 | 0.0471 |

## Supplementary table 3. Internal genetic correlations between treatment seeking phenotypes

| **Treatment phenotype 1** | **Treatment phenotype 2** | **Sample 1** | **Sample 2** | **rg** | **se** | **z** | **p** |
| --- | --- | --- | --- | --- | --- | --- | --- |
| Formal treatment-seeking | Formal treatment-seeking | Full | Females | .9842 | .0083 | 118.8656 | 0 |
| Formal treatment-seeking | Formal treatment-seeking | Full | Males | .9635 | .0151 | 63.7486 | 0 |
| Formal treatment-seeking | Formal treatment-seeking | Males | Females | .8994 | .0435 | 2.678 | 5.46E-95 |
| Broad treatment-seeking | Broad treatment-seeking | Females | Full | 1.0058 | .0503 | 2.0119 | 4.34E-89 |
| Self-help | Self-help | Females | Full | 1.0983 | .0659 | 16.6607 | 2.53E-62 |
| Self-medication | Self-medication | Females | Full | .9933 | .0698 | 14.2395 | 5.21E-46 |
| Broad treatment-seeking | Broad treatment-seeking | Controls | Full | .9071 | .0662 | 13.6943 | 1.10E-42 |
| Broad treatment-seeking | Broad treatment-seeking | Males | Full | .9888 | .0795 | 12.4375 | 1.63E-35 |
| Formal treatment-seeking | Broad treatment-seeking | Full | Full | .9877 | .0836 | 11.8095 | 3.49E-32 |
| Formal treatment-seeking | Broad treatment-seeking | Females | Full | .9847 | .0869 | 11.3347 | 8.84E-30 |
| Self-medication | Self-medication | Males | Full | .9941 | .0892 | 11.1454 | 7.54E-29 |
| Self-help | Self-help | Cases | Full | .9175 | .0952 | 9.6413 | 5.35E-22 |
| Formal treatment-seeking | Broad treatment-seeking | Males | Full | .93 | .0975 | 9.5354 | 1.49E-21 |
| Broad treatment-seeking | Broad treatment-seeking | Females | Controls | .9083 | .1168 | 7.7793 | 7.29E-15 |
| Formal treatment-seeking | Broad treatment-seeking | Full | Females | 1.1154 | .1496 | 7.4533 | 9.10E-14 |
| Formal treatment-seeking | Broad treatment-seeking | Females | Females | 1.1193 | .1534 | 7.296 | 2.96E-13 |
| Self-medication | Self-medication | Cases | Full | .9631 | .1467 | 6.5655 | 5.18E-11 |
| Formal treatment-seeking | Broad treatment-seeking | Males | Females | 1.0464 | .1633 | 6.4073 | 1.48E-10 |
| Formal treatment-seeking | Broad treatment-seeking | Full | Males | .83 | .13 | 6.3865 | 1.70E-10 |
| Self-help | Self-help | Females | Cases | .985 | .1577 | 6.247 | 4.18E-10 |
| Broad treatment-seeking | Formal treatment-seeking | Males | Females | .813 | .1355 | 5.9993 | 1.98E-09 |
| Broad treatment-seeking | Broad treatment-seeking | Males | Controls | .9019 | .1508 | 5.9799 | 2.23E-09 |
| Self-medication | Self-medication | Controls | Full | .8109 | .1359 | 5.9659 | 2.43E-09 |
| Formal treatment-seeking | Broad treatment-seeking | Full | Controls | .7483 | .1274 | 5.8743 | 4.25E-09 |
| Formal treatment-seeking | Broad treatment-seeking | Males | Males | .7921 | .1392 | 5.6903 | 1.27E-08 |
| Formal treatment-seeking | Broad treatment-seeking | Females | Controls | .705 | .1299 | 5.4261 | 5.76E-08 |
| Formal treatment-seeking | Self-help | Full | Full | .4813 | .0912 | 5.279 | 1.30E-07 |
| Broad treatment-seeking | Broad treatment-seeking | Males | Controls | .7666 | .1464 | 5.2354 | 1.65E-07 |
| Broad treatment-seeking | Broad treatment-seeking | Cases | Full | .8059 | .1573 | 5.1237 | 3.00E-07 |
| Formal treatment-seeking | Self-help | Females | Full | .5039 | .1011 | 4.9849 | 6.20E-07 |
| Self-medication | Self-medication | Females | Cases | .8754 | .1831 | 4.7812 | 1.74E-06 |
| Self-medication | Self-medication | Males | Controls | .8621 | .2195 | 3.9277 | 8.58E-05 |
| Broad treatment-seeking | Broad treatment-seeking | Females | Cases | .8219 | .2103 | 3.9089 | 9.27E-05 |
| Broad treatment-seeking | Broad treatment-seeking | Males | Females | .9929 | .2543 | 3.9052 | 9.41E-05 |
| Formal treatment-seeking | Broad treatment-seeking | Full | Cases | .7886 | .2045 | 3.8565 | 1.00E-04 |
| Self-medication | Self-help | Full | Full | .6324 | .1696 | 3.7297 | 2.00E-04 |
| Formal treatment-seeking | Self-medication | Females | Full | .313 | .0854 | 3.6654 | 2.00E-04 |
| Formal treatment-seeking | Broad treatment-seeking | Females | Cases | .8347 | .2204 | 3.7869 | 2.00E-04 |
| Formal treatment-seeking | Self-medication | Full | Full | .2895 | .0775 | 3.735 | 2.00E-04 |
| Formal treatment-seeking | Self-help | Males | Full | .4051 | .1123 | 3.6082 | 3.00E-04 |
| Formal treatment-seeking | Self-medication | Full | Females | .3917 | .1073 | 3.6521 | 3.00E-04 |
| Self-medication | Self-medication | Males | Cases | 1.0347 | .2914 | 3.5511 | 4.00E-04 |
| Formal treatment-seeking | Broad treatment-seeking | Males | Cases | .6914 | .1969 | 3.5121 | 4.00E-04 |
| Formal treatment-seeking | Self-help | Full | Cases | .4319 | .1209 | 3.5737 | 4.00E-04 |
| Formal treatment-seeking | Self-medication | Females | Females | .3833 | .1117 | 3.4325 | 6.00E-04 |
| Formal treatment-seeking | Self-help | Full | Females | .4296 | .1251 | 3.4333 | 6.00E-04 |
| Broad treatment-seeking | Self-help | Full | Full | .5026 | .1496 | 3.3592 | 8.00E-04 |
| Broad treatment-seeking | Broad treatment-seeking | Males | Cases | .761 | .232 | 3.2802 | .001 |
| Formal treatment-seeking | Self-help | Females | Cases | .4075 | .127 | 3.209 | .0013 |
| Self-medication | Self-medication | Males | Females | .9776 | .3136 | 3.1171 | .0018 |
| Formal treatment-seeking | Self-help | Females | Females | .4361 | .1434 | 3.041 | .0024 |
| Self-medication | Self-medication | Females | Controls | .7656 | .2532 | 3.0242 | .0025 |
| Formal treatment-seeking | Self-help | Males | Cases | .4404 | .1472 | 2.9921 | .0028 |
| Self-medication | Self-help | Females | Full | .7167 | .2545 | 2.816 | .0049 |
| Broad treatment-seeking | Self-help | Females | Full | .5469 | .1958 | 2.7932 | .0052 |
| Broad treatment-seeking | Self-medication | Full | Full | .3349 | .1219 | 2.7477 | .006 |
| Self-medication | Self-help | Full | Females | .5915 | .2179 | 2.715 | .0066 |
| Formal treatment-seeking | Self-help | Males | Females | .374 | .1381 | 2.7091 | .0067 |
| Broad treatment-seeking | Self-help | Full | Females | .5516 | .2043 | 2.7001 | .0069 |
| Broad treatment-seeking | Self-medication | Females | Full | .4372 | .1671 | 2.6165 | .0089 |
| Formal treatment-seeking | Self-medication | Males | Females | .3561 | .1365 | 2.6081 | .0091 |
| Broad treatment-seeking | Self-help | Controls | Full | .609 | .2375 | 2.5648 | .0103 |
| Self-medication | Self-help | Males | Full | .5202 | .2109 | 2.4661 | .0137 |

# Formal treatment-seeking (replication analyses)

The whole UK Biobank sample (N~500,000) were also asked the question: *“Have you ever seen a GP / psychiatrist for nerves, anxiety, tension or depression?”.* From this data, we defined a **formal** treatment-seeking phenotype, i.e. participants who reported on seeking help from their doctor or a psychiatrist. Data were available for 391,213 participants reporting on **formal** treatment seeking. Of which, 31% report seeking treatment within their lifetimes. Stratified analyses based on “need” (i.e. cases/controls) were not possible in the formal treatment-seeking phenotype, because symptom level and diagnostic data were not available for most of these participants. We excluded the participants from our main analyses (referred to now as **broad** treatment-seeking) from this secondary, **formal** treatment-seeking phenotype to provide an independent analysis sample. We saw this as an opportunity to test the reproducibility of the primary treatment-seeking analyses in a much larger sample. We use the broad/formal distinction here because, in our main analyses, help can be sought from any ‘professional’. Whereas, this secondary analysis assesses help sought specifically from a doctor.

## Supplementary table 4. Phenotype distribution of primary treatment-seeking phenotype and supplementary, formal treatment-seeking phenotype

|  |  |  | **Lifetime diagnosis** | | **Sex** | |
| --- | --- | --- | --- | --- | --- | --- |
|  |  | ***N*** | ***No*** | ***Yes*** | ***Female*** | ***Male*** |
| Broad treatment-seeking*^(1)^* | ***No*** | 23,362 | 16,789 | 4,222 | 12,528 | 10,834 |
|  | ***Yes*** | 48,054 | 14,700 | 25,810 | 32,706 | 15,348 |
|  | ***Prev.*** ^(^*****^)^ | 0.67 | 0.47 | 0.86 | 0.72 | 0.59 |
| Formal treatment-seeking*^(2)^* | ***No*** | 268,184 | - | - | 129,936 | 146,412 |
|  | ***Yes*** | 123,029 | - | - | 80,868 | 46,458 |
|  | ***Prev.*** | 0.31 |  |  | 0.38 | 0.24 |
| *^(1)^ Broad treatment-seeking, i.e. the treatment-seeking phenotype examined in the main analysis, treatment-seeking from a professional for anxiety or depression; ^(2)^ Formal treatment-seeking from a GP or Psychiatrist for anxiety, depression, stress or tension; All other phenotypes are in response to symptoms of anxiety or depression* | | | | | | |

As before, we estimated the heritability of **formal** treatment-seeking. Here, estimates were obtained in the full sample (h^2^_g_ *_(obs)_*=6.4% se=.3%), and in males (h^2^_g_ *_(obs)_*=5.6% se=.4%) and females (h^2^_g_ *_(obs)_*=7.9% se=.5%). In this instance, heritability estimates are significantly different between males and females (block jackknife estimate=-3%, se=.5%, p=1.4 x 10^-8^). As expected, standard errors in this sample are smaller than in the previous analysis of treatment seeking. However, estimates are also significantly different between the samples (MHQ v UKB; Full sample: block jackknife estimate=-.02, se=.01, p=.01; Females: est=-.04, se=.01, p=2.2e-05; Males: est=.01, se=.02, p=.49).

## Supplementary Figure 7 (a) Observed common genetic variant heritability estimates of treatment-seeking in the primary (broad) and secondary cohort (formal), and stratified by sex (b) Common genetic variant heritability curves: heritability estimates of treatment-seeking converted to the liability scale

(a)


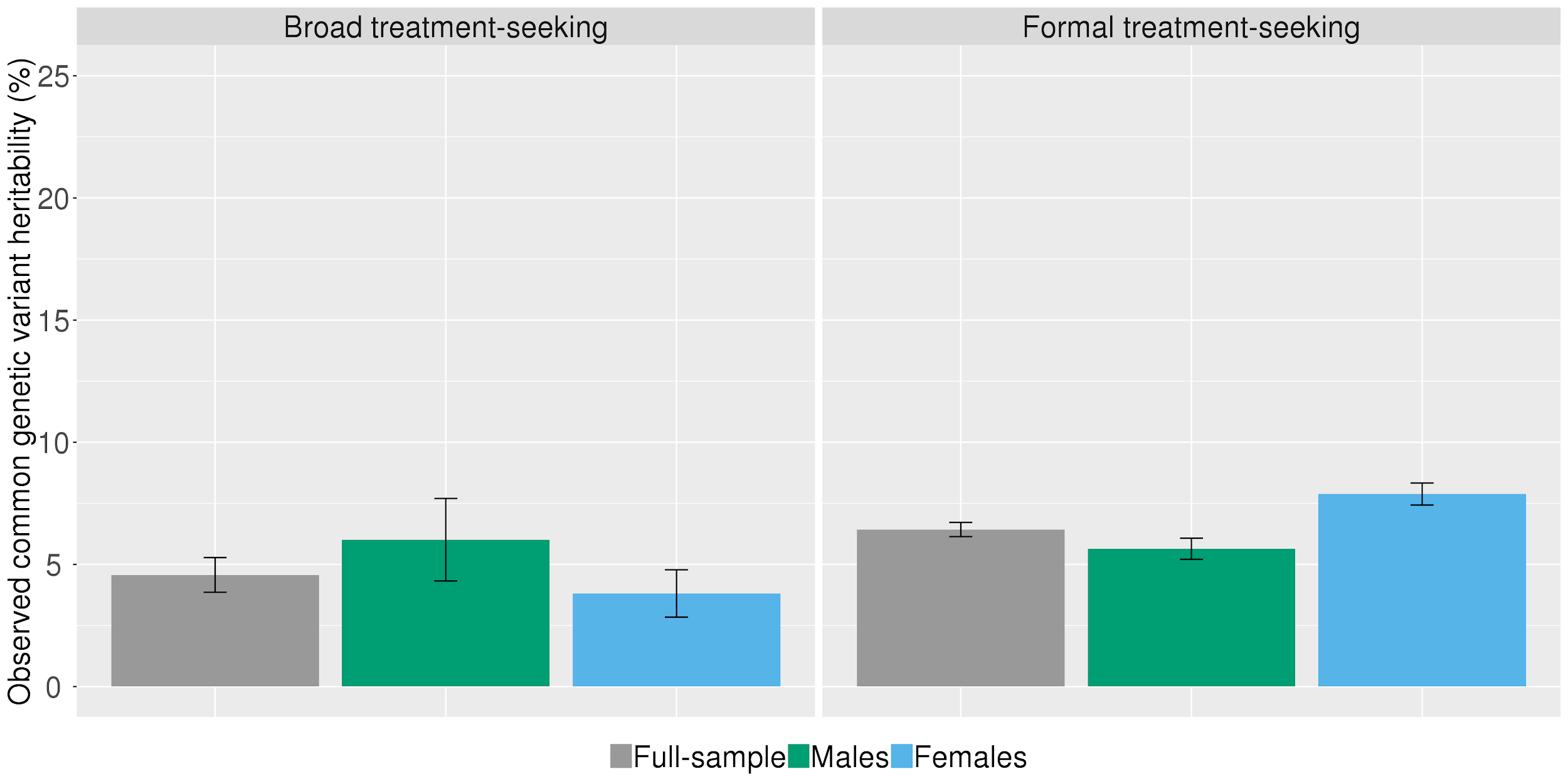


(b)


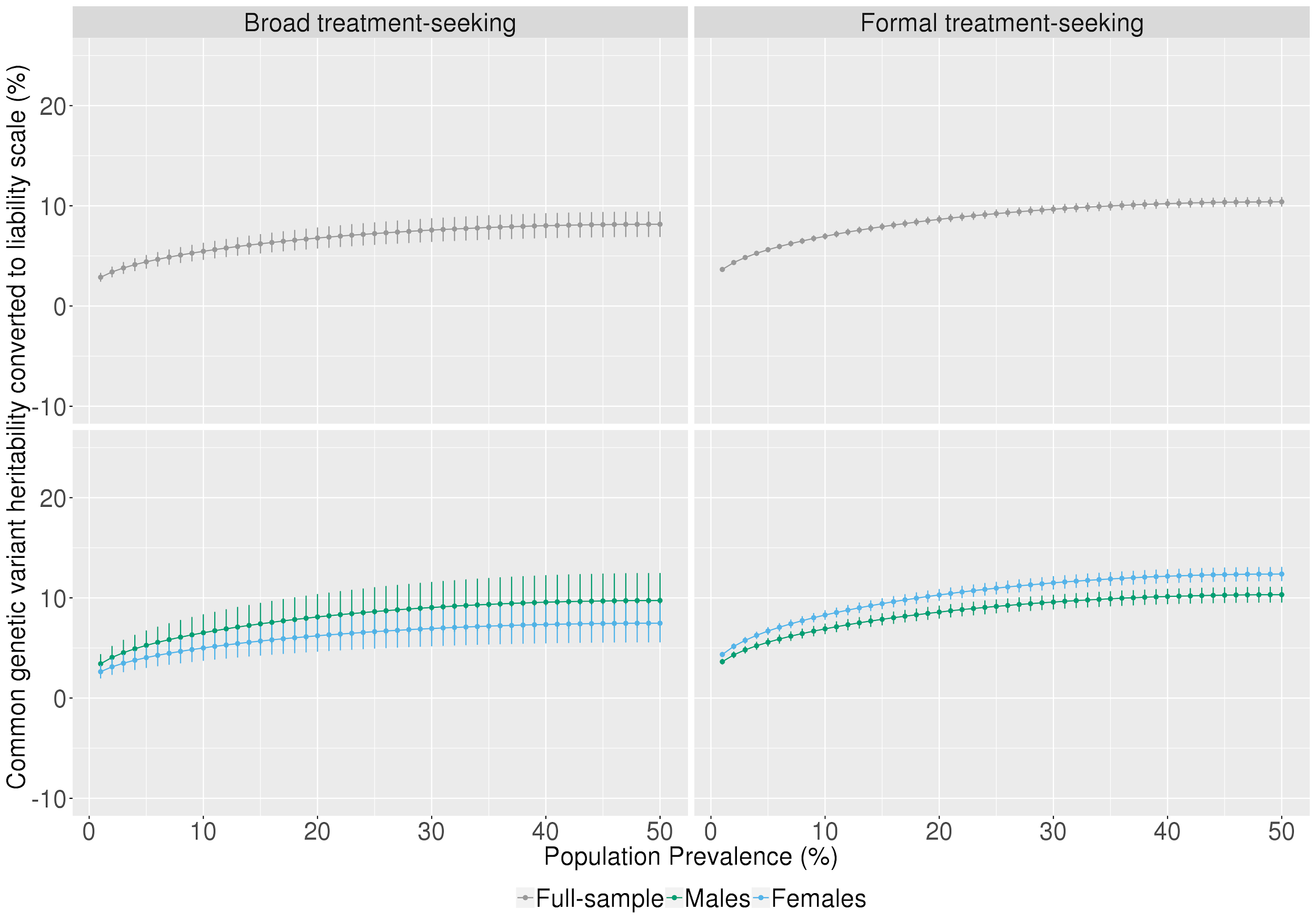


Broad treatment-seeking in the primary sample and in the second sample have a genetic correlation that is not significantly different from 1 (r_g_=.99, se=.08). Indeed, all of the genetic correlations between treatment-seeking and disorders, behavioural and cognitive traits from our primary analysis are replicated in this secondary analysis. Additional significant genetic correlations were also detected with borderline personality disorder (r_g_=0.57,se=0.11), social deprivation (r_g_=0.44, se=0.06), and intelligence (r_g_=-017,se=0.02).

## Supplementary Figure 8. Genetic correlations of treatment-seeking and external traits stratified by sex in the primary sample (**broad treatment-seeking)** and the secondary sample (**formal treatment-seeking)**

**
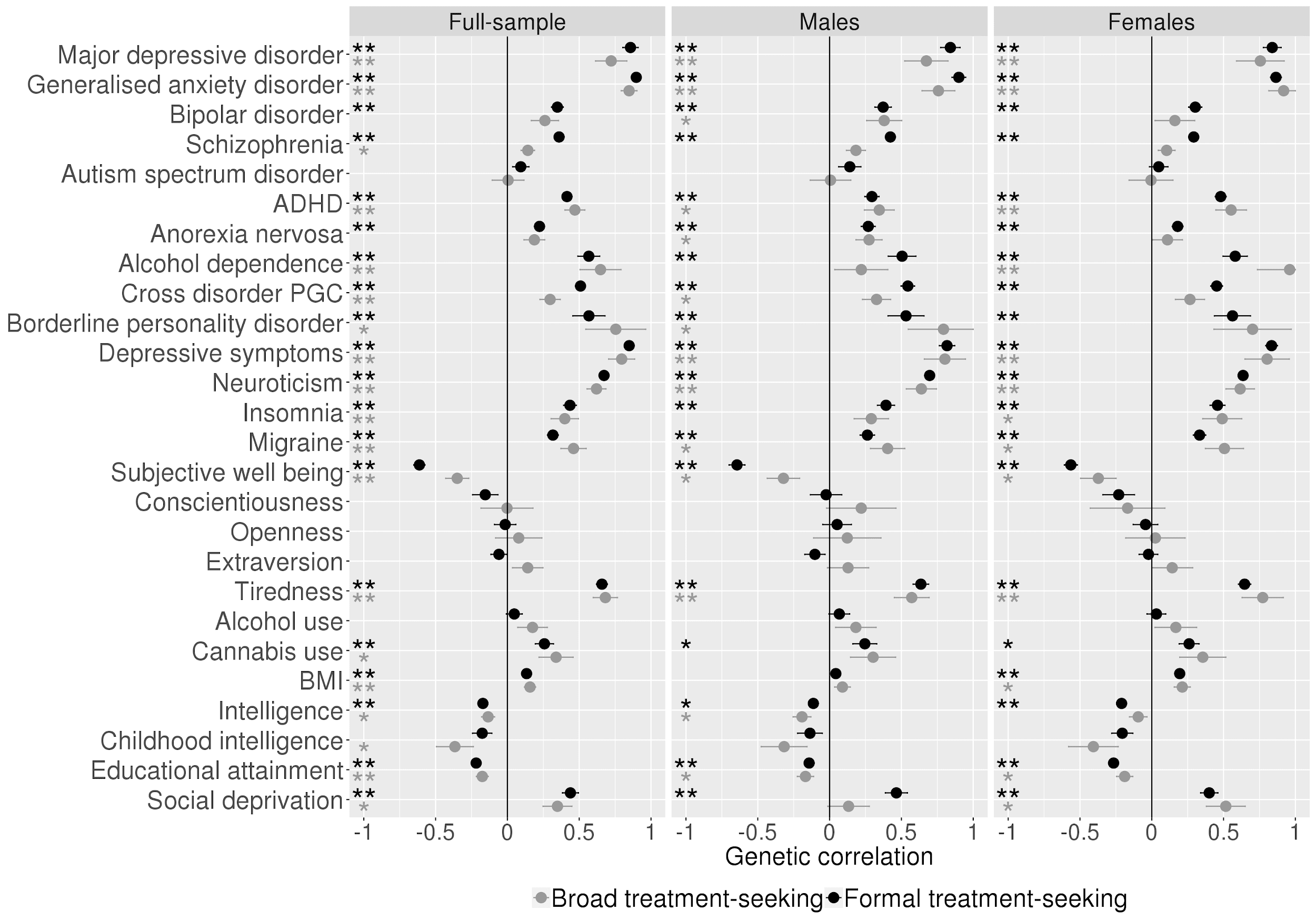
**

## Supplementary Figure 9. Manhattan and QQ plots for GWAS of Formal treatment-seeking

a. Full sample (n=391,213):


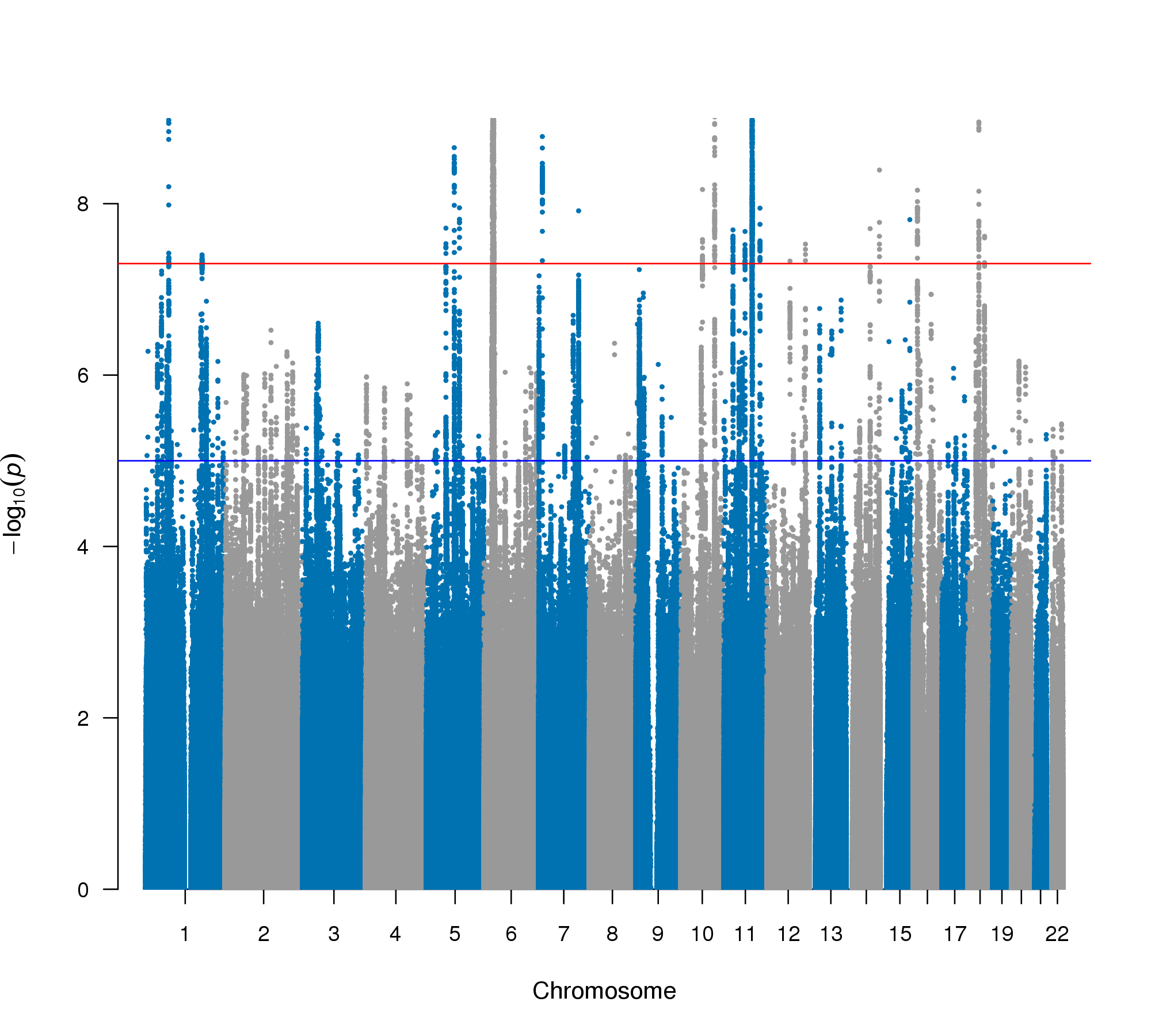

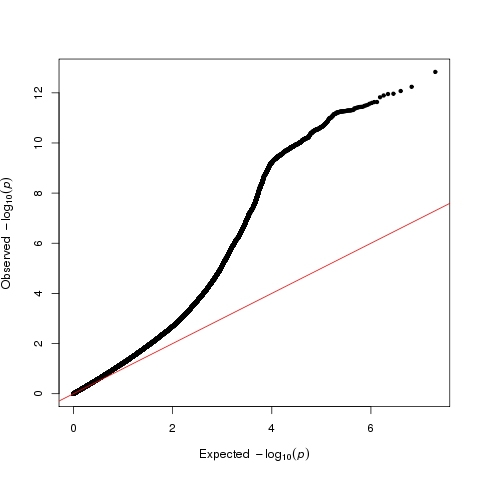


b. Males (n=180,409):


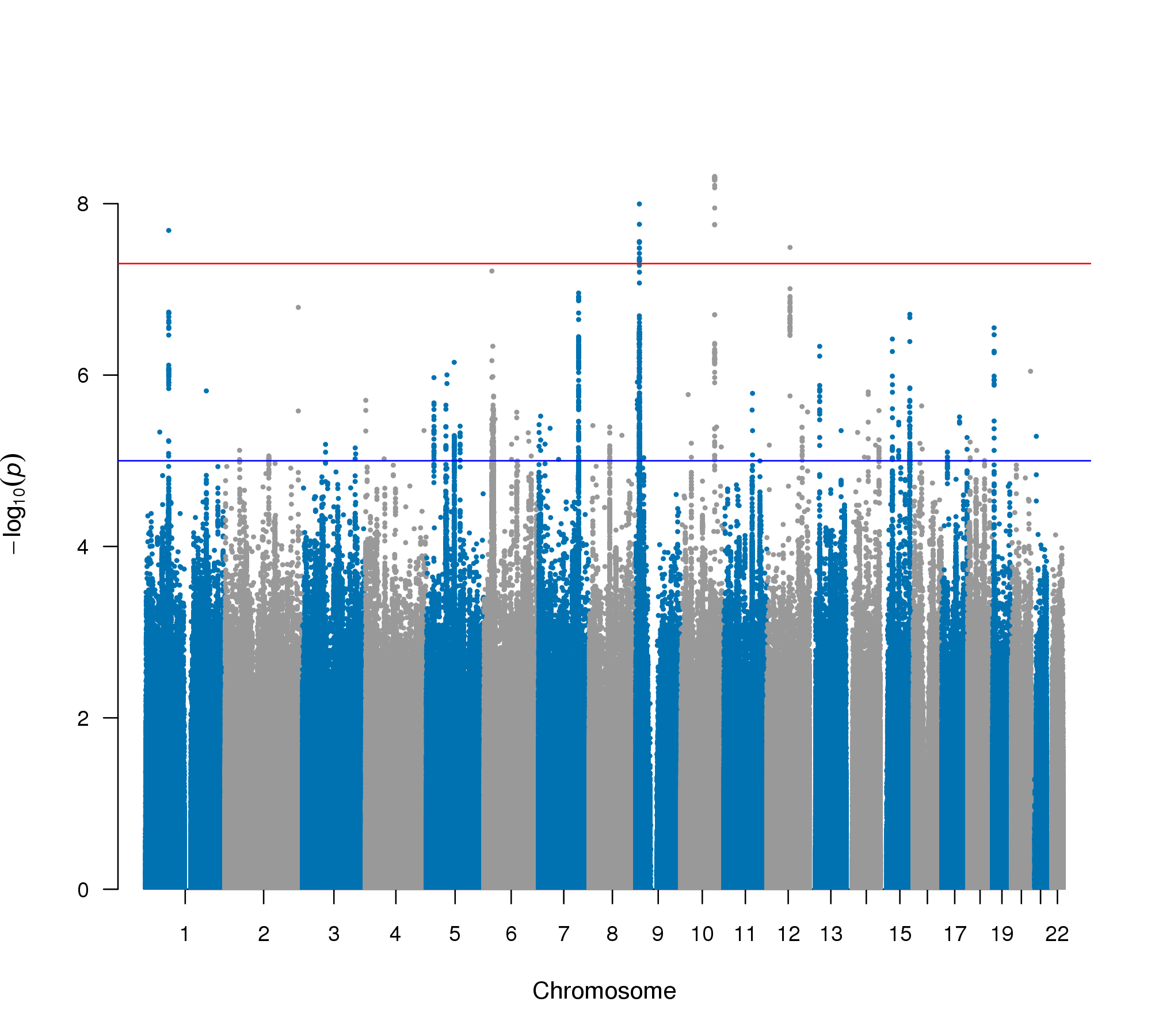

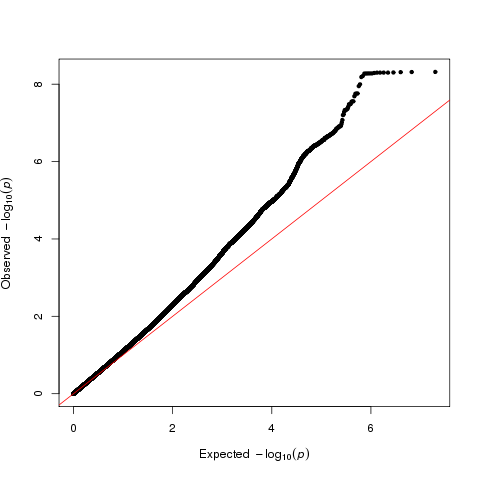


c. Females (n=210,804):


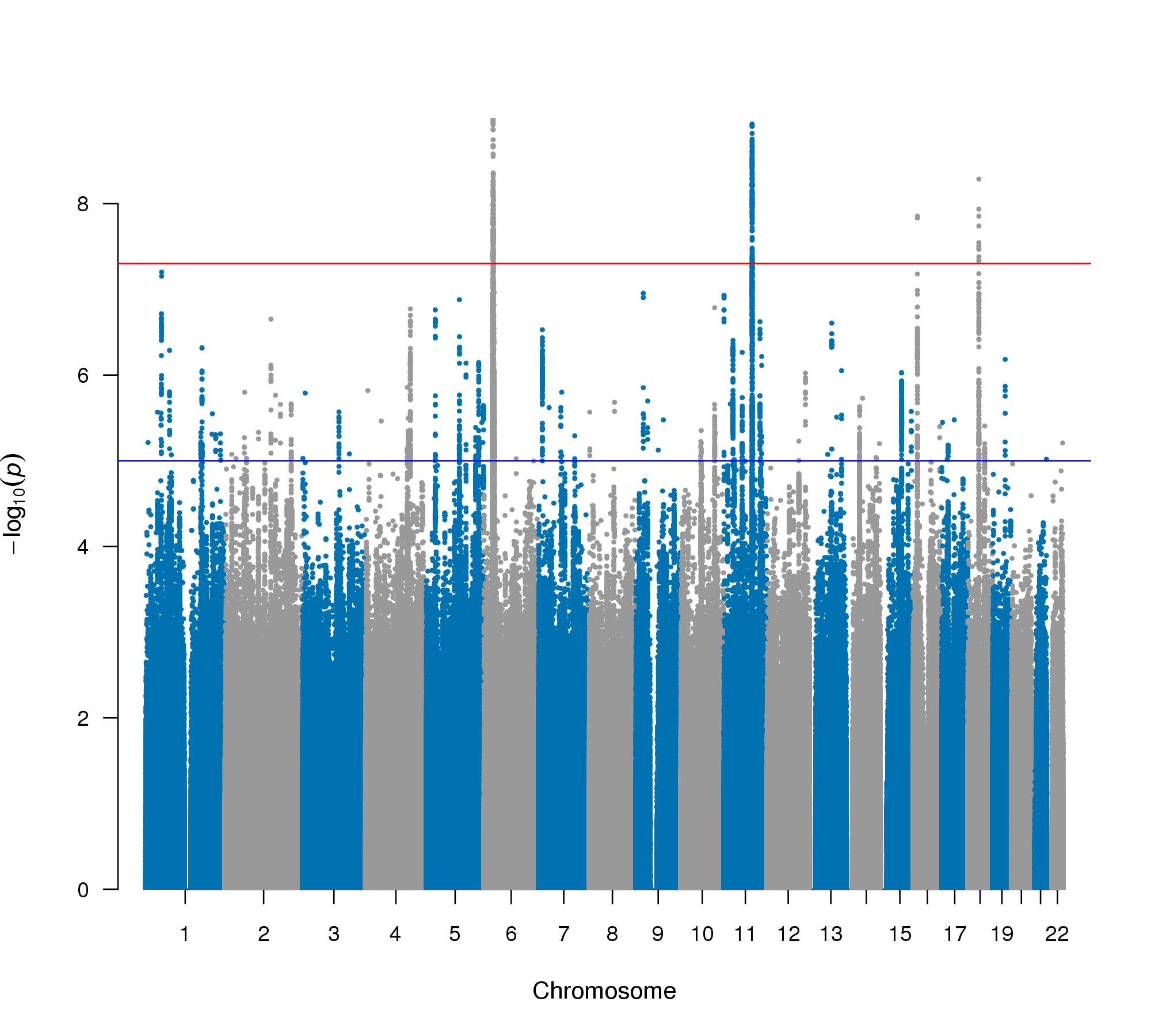

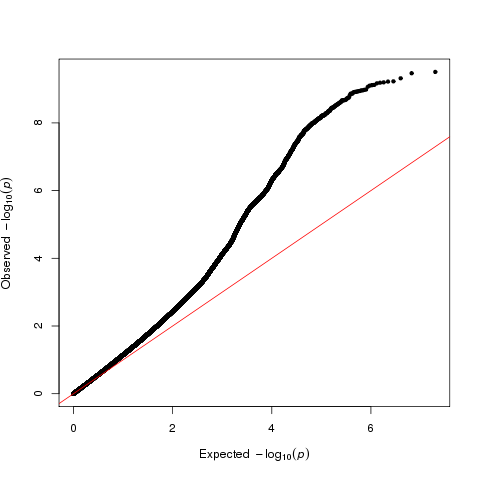


# Supplementary References

[Abraham, Gad, Yixuan Qiu, and Michael Inouye. 2017. “FlashPCA2: Principal Component Analysis of Biobank-Scale Genotype Datasets.” *Bioinformatics*  33 (17): 2776–78.](http://paperpile.com/b/ir95Fc/ykhr)

[Allen, Naomi E., Cathie Sudlow, Tim Peakman, Rory Collins, and UK Biobank. 2014. “UK Biobank Data: Come and Get It.” *Science Translational Medicine* 6 (224): 224ed4.](http://paperpile.com/b/ir95Fc/xuln)

[Autism Spectrum Disorders Working Group of The Psychiatric Genomics Consortium. 2017. “Meta-Analysis of GWAS of over 16,000 Individuals with Autism Spectrum Disorder Highlights a Novel Locus at 10q24.32 and a Significant Overlap with Schizophrenia.” *Molecular Autism* 8 (1): 21.](http://paperpile.com/b/ir95Fc/Ro0Ns)

[Benyamin, B., Bst Pourcain, O. S. Davis, G. Davies, N. K. Hansell, M-J A. Brion, R. M. Kirkpatrick, et al. 2014. “Childhood Intelligence Is Heritable, Highly Polygenic and Associated with FNBP1L.” *Molecular Psychiatry* 19 (2): 253–58.](http://paperpile.com/b/ir95Fc/O6VvK)

[Bulik, Cynthia, Laramie Duncan, Gerome Breen, and PGC_AN Working Group. 2017. “The PGC Gwas Meta-Analysis of Anorexia Nervosa: SNP Heritability, Genetic Correlations, And Snp Results.” *European Neuropsychopharmacology: The Journal of the European College of Neuropsychopharmacology* 27 (January): S360–61.](http://paperpile.com/b/ir95Fc/XUEkd)

[Bulik-Sullivan, Brendan K., Po-Ru Loh, Hilary K. Finucane, Stephan Ripke, Jian Yang, Schizophrenia Working Group of the Psychiatric Genomics Consortium, Nick Patterson, Mark J. Daly, Alkes L. Price, and Benjamin M. Neale. 2015. “LD Score Regression Distinguishes Confounding from Polygenicity in Genome-Wide Association Studies.” *Nature Genetics* 47 (3): 291–95.](http://paperpile.com/b/ir95Fc/H6Yc)

[Bycroft, Clare, Colin Freeman, Desislava Petkova, Gavin Band, Lloyd T. Elliott, Kevin Sharp, Allan Motyer, et al. 2017. “Genome-Wide Genetic Data on ~500,000 UK Biobank Participants.” *bioRxiv*.](http://paperpile.com/b/ir95Fc/WIdz) <http://www.biorxiv.org/content/early/2017/07/20/166298>[.](http://paperpile.com/b/ir95Fc/WIdz)

[Cross-Disorder Group of the Psychiatric Genomics Consortium. 2013. “Identification of Risk Loci with Shared Effects on Five Major Psychiatric Disorders: A Genome-Wide Analysis.” *The Lancet* 381 (9875): 1371–79.](http://paperpile.com/b/ir95Fc/0H7B8)

[Deary, V., S. P. Hagenaars, S. E. Harris, W. D. Hill, G. Davies, D. C. M. Liewald, International Consortium for Blood Pressure GWAS, et al. 2018. “Genetic Contributions to Self-Reported Tiredness.” *Molecular Psychiatry* 23 (3): 609–20.](http://paperpile.com/b/ir95Fc/SPwbE)

[Demontis, Ditte, Raymond K. Walters, Joanna Martin, Manuel Mattheisen, Thomas D. Als, Esben Agerbo, Gísli Baldursson, et al. 2018. “Discovery of the First Genome-Wide Significant Risk Loci for Attention Deficit/hyperactivity Disorder.” *Nature Genetics*, November. https://doi.org/](http://paperpile.com/b/ir95Fc/UhH4a)[10.1038/s41588-018-0269-7](http://dx.doi.org/10.1038/s41588-018-0269-7)[.](http://paperpile.com/b/ir95Fc/UhH4a)

[Falconer, Douglas S. 1965. “The Inheritance of Liability to Certain Diseases, Estimated from the Incidence among Relatives.” *Annals of Human Genetics* 29: 51–76.](http://paperpile.com/b/ir95Fc/iu0OV)

[Gormley, Padhraig, Verneri Anttila, Bendik S. Winsvold, Priit Palta, Tonu Esko, Tune H. Pers, Kai-How Farh, et al. 2016. “Meta-Analysis of 375,000 Individuals Identifies 38 Susceptibility Loci for Migraine.” *Nature Genetics* 48 (8): 856–66.](http://paperpile.com/b/ir95Fc/RD9TI)

[Hammerschlag, Anke R., Sven Stringer, Christiaan A. de Leeuw, Suzanne Sniekers, Erdogan Taskesen, Kyoko Watanabe, Tessa F. Blanken, et al. 2017. “Genome-Wide Association Analysis of Insomnia Complaints Identifies Risk Genes and Genetic Overlap with Psychiatric and Metabolic Traits.” *Nature Genetics* 49 (11): 1584–92.](http://paperpile.com/b/ir95Fc/n9wxM)

[Hill, W. David, Saskia P. Hagenaars, Riccardo E. Marioni, Sarah E. Harris, David C. M. Liewald, Gail Davies, Aysu Okbay, Andrew M. McIntosh, Catharine R. Gale, and Ian J. Deary. 2016. “Molecular Genetic Contributions to Social Deprivation and Household Income in UK Biobank.” *Current Biology: CB* 26 (22): 3083–89.](http://paperpile.com/b/ir95Fc/Zqlxp)

[Lee, James J., Robbee Wedow, Aysu Okbay, Edward Kong, Omeed Maghzian, Meghan Zacher, Tuan Anh Nguyen-Viet, et al. 2018. “Gene Discovery and Polygenic Prediction from a Genome-Wide Association Study of Educational Attainment in 1.1 Million Individuals.” *Nature Genetics* 50 (8): 1112–21.](http://paperpile.com/b/ir95Fc/4bpwk)

[Manichaikul, Ani, Josyf C. Mychaleckyj, Stephen S. Rich, Kathy Daly, Michèle Sale, and Wei-Min Chen. 2010. “Robust Relationship Inference in Genome-Wide Association Studies.” *Bioinformatics*  26 (22): 2867–73.](http://paperpile.com/b/ir95Fc/NSdB)

[McCarthy, Shane, Sayantan Das, Warren Kretzschmar, Olivier Delaneau, Andrew R. Wood, Alexander Teumer, Hyun Min Kang, et al. 2016. “A Reference Panel of 64,976 Haplotypes for Genotype Imputation.” *Nature Genetics* 48 (10): 1279–83.](http://paperpile.com/b/ir95Fc/iXWO)

[Moor, M. H. M. de, P. T. Costa, A. Terracciano, R. F. Krueger, E. J. C. de Geus, T. Toshiko, B. W. J. H. Penninx, et al. 2010. “Meta-Analysis of Genome-Wide Association Studies for Personality.” *Molecular Psychiatry* 17 (December): 337.](http://paperpile.com/b/ir95Fc/82zqH)

[Okbay, Aysu, Bart M. L. Baselmans, Jan-Emmanuel De Neve, Patrick Turley, Michel G. Nivard, Mark Alan Fontana, S. Fleur W. Meddens, et al. 2016. “Genetic Variants Associated with Subjective Well-Being, Depressive Symptoms, and Neuroticism Identified through Genome-Wide Analyses.” *Nature Genetics* 48 (6): 624–33.](http://paperpile.com/b/ir95Fc/y2cFr)

[Psychiatric GWAS Consortium Bipolar Disorder Working Group. 2011. “Large-Scale Genome-Wide Association Analysis of Bipolar Disorder Identifies a New Susceptibility Locus near ODZ4.” *Nature Genetics* 43 (10): 977–83.](http://paperpile.com/b/ir95Fc/LQH7v)

[Purves, Kirstin, Jonathan R. I. Coleman, Chris Rayner, Jack M. Hettema, Jurgen Deckert, Andrew M. McIntosh, Kristin Nicodemus, Gerome Breen, and Thalia C. Eley. 2017. “The Common Genetic Architecture of Anxiety Disorders.” *BioRxiv*. https://doi.org/](http://paperpile.com/b/ir95Fc/GcAbC)[10.1101/203844](http://dx.doi.org/10.1101/203844)[.](http://paperpile.com/b/ir95Fc/GcAbC)

[Ripke, Stephan, Benjamin M. Neale, Aiden Corvin, James T. R. Walters, Kai-How Farh, Peter A. Holmans, Phil Lee, et al. 2014. “Biological Insights from 108 Schizophrenia-Associated Genetic Loci.” *Nature* 511 (7510): 421–27.](http://paperpile.com/b/ir95Fc/Z9Ctl)

[Savage, Jeanne E., Philip R. Jansen, Sven Stringer, Kyoko Watanabe, Julien Bryois, Christiaan A. de Leeuw, Mats Nagel, et al. 2018. “Genome-Wide Association Meta-Analysis in 269,867 Individuals Identifies New Genetic and Functional Links to Intelligence.” *Nature Genetics*, June. https://doi.org/](http://paperpile.com/b/ir95Fc/p2AYO)[10.1038/s41588-018-0152-6](http://dx.doi.org/10.1038/s41588-018-0152-6)[.](http://paperpile.com/b/ir95Fc/p2AYO)

[Schumann, Gunter, Chunyu Liu, Paul O’Reilly, He Gao, Parkyong Song, Bing Xu, Barbara Ruggeri, et al. 2016. “KLB Is Associated with Alcohol Drinking, and Its Gene Product β-Klotho Is Necessary for FGF21 Regulation of Alcohol Preference.” *Proceedings of the National Academy of Sciences of the United States of America* 113 (50): 14372–77.](http://paperpile.com/b/ir95Fc/UcTYP)

[Stringer, S., C. C. Minică, K. J. H. Verweij, H. Mbarek, M. Bernard, J. Derringer, K. R. van Eijk, et al. 2016. “Genome-Wide Association Study of Lifetime Cannabis Use Based on a Large Meta-Analytic Sample of 32 330 Subjects from the International Cannabis Consortium.” *Translational Psychiatry* 6 (March): e769.](http://paperpile.com/b/ir95Fc/jctJU)

[UK10K Consortium, Klaudia Walter, Josine L. Min, Jie Huang, Lucy Crooks, Yasin Memari, Shane McCarthy, et al. 2015. “The UK10K Project Identifies Rare Variants in Health and Disease.” *Nature* 526 (7571): 82–90.](http://paperpile.com/b/ir95Fc/DIfJ)

[Visscher, Peter M., William G. Hill, and Naomi R. Wray. 2008. “Heritability in the Genomics Era--Concepts and Misconceptions.” *Nature Reviews. Genetics* 9 (4): 255–66.](http://paperpile.com/b/ir95Fc/LUhZp)

[Walters, Raymond K., Mark J. Adams, Amy E. Adkins, Fazil Aliev, Silviu-Alin Bacanu, Anthony Batzler, Sarah Bertelsen, et al. 2018. “Trans-Ancestral GWAS of Alcohol Dependence Reveals Common Genetic Underpinnings with Psychiatric Disorders.” *bioRxiv*. https://doi.org/](http://paperpile.com/b/ir95Fc/aauI3)[10.1101/257311](http://dx.doi.org/10.1101/257311)[.](http://paperpile.com/b/ir95Fc/aauI3)

[Warren, Helen R., Evangelos Evangelou, Claudia P. Cabrera, He Gao, Meixia Ren, Borbala Mifsud, Ioanna Ntalla, et al. 2017. “Genome-Wide Association Analysis Identifies Novel Blood Pressure Loci and Offers Biological Insights into Cardiovascular Risk.” *Nature Genetics* 49 (3): 403–15.](http://paperpile.com/b/ir95Fc/AbCu)

[Witt, S. H., F. Streit, M. Jungkunz, J. Frank, S. Awasthi, C. S. Reinbold, J. Treutlein, et al. 2017. “Genome-Wide Association Study of Borderline Personality Disorder Reveals Genetic Overlap with Bipolar Disorder, Major Depression and Schizophrenia.” *Translational Psychiatry* 7 (6): e1155.](http://paperpile.com/b/ir95Fc/kwhDU)

[Wray, Naomi R., Stephan Ripke, Manuel Mattheisen, Maciej Trzaskowski, Enda M. Byrne, Abdel Abdellaoui, Mark J. Adams, et al. 2018. “Genome-Wide Association Analyses Identify 44 Risk Variants and Refine the Genetic Architecture of Major Depression.” *Nature Genetics* 50 (5): 668–81.](http://paperpile.com/b/ir95Fc/c8u3s)

[Yengo, Loic, Julia Sidorenko, Kathryn E. Kemper, Zhili Zheng, Andrew R. Wood, Michael N. Weedon, Timothy M. Frayling, et al. 2018. “Meta-Analysis of Genome-Wide Association Studies for Height and Body Mass Index in ~700,000 Individuals of European Ancestry.” *bioRxiv*. https://doi.org/](http://paperpile.com/b/ir95Fc/og6jh)[10.1101/274654](http://dx.doi.org/10.1101/274654)[.](http://paperpile.com/b/ir95Fc/og6jh)
